# Tailored Porous Carbon Xerogels for Fe-N-C Catalysts in Proton Exchange Membrane Fuel Cells

**DOI:** 10.3390/nano14010014

**Published:** 2023-12-20

**Authors:** Laura Álvarez-Manuel, Cinthia Alegre, David Sebastián, Pedro F. Napal, María Jesús Lázaro

**Affiliations:** Instituto de Carboquímica, Consejo Superior de Investigaciones Científicas, 50018 Zaragoza, Spain; lalvarez@icb.csic.es (L.Á.-M.); dsebastian@icb.csic.es (D.S.); pnapal@icb.csic.es (P.F.N.)

**Keywords:** carbon xerogels, Fe-N-C catalysts, oxygen reduction reaction, fuel cells

## Abstract

Atomically dispersed Fe-N-C catalysts for the oxygen reduction reaction (ORR) have been synthesized with a template-free method using carbon xerogels (CXG) as a porous matrix. The porosity of the CXGs is easily tunable through slight variations in the synthesis procedure. In this work, CXGs are prepared by formaldehyde and resorcinol polymerization, modifying the pH during the process. Materials with a broad range of porous structures are obtained: from non-porous to micro-/meso-/macroporous materials. The porous properties of CXG have a direct effect on Fe-N-CXG activity against ORR in an acidic medium (0.5 M H_2_SO_4_). Macropores and wide mesopores are vital to favor the mass transport of reagents to the active sites available in the micropores, while narrower mesopores can generate additional tortuosity. The role of microporosity is investigated by comparing two Fe-N-C catalysts using the same CXG as the matrix but following a different Fe and N doping procedure. In one case, the carbonization of CXG occurs rapidly and simultaneously with Fe and N doping, whereas in the other case it proceeds slowly, under controlled conditions and before the doping process, resulting in the formation of more micropores and active sites and achieving higher activity in a three-electrode cell and a better durability during fuel cell measurements. This work proves the feasibility of the template-free method using CXG as a carbon matrix for Fe-N-C catalysts, with the novelty of the controlled porous properties of the carbon material and its effect on the catalytic activity of the Fe-N-C catalyst. Moreover, the results obtained highlight the importance of the carbon matrix’s porous structure in influencing the activity of Fe-N-C catalysts against ORR.

## 1. Introduction

Although fuel cells have been considered a promising solution for current energy demands, their high production cost remains a challenge. Currently, the US Department of Energy (US DOE) [1] reports that catalysts account for 59% of the fuel cell stack cost. To reduce this cost, extensive research has been conducted to develop cost-effective catalysts without precious metals in their formulation [2,3,4,5].

Among various non-precious metal cathode catalysts, Fe-N-C catalysts have shown great potential, demonstrating superior activity and durability in comparison to other centers such as Zn, Mn, or Co [6]. Moreover, Fe is even more interesting as a catalyst due to its low toxicity, recyclability, and high abundance [7,8]. Although the exact character of the Fe-N-C catalysts’ active sites is currently under debate, some consensus has been achieved from the extensive literature on Fe-N-C catalysts published in recent years. First, Fe-N_4_ atomically dispersed sites are generally agreed to be the most potent and discerning centers for the four-electron ORR process in acidic media [9,10,11,12]. Second, it is known that the porosity characteristics of catalysts have a vital role in defining their catalytic efficiency [13].

The Fe-N active sites must be embedded in a porous carbon hierarchical structure composed of interconnected macropores, mesopores, and micropores to effectively channel reactants to the active sites, which are situated in the micropores [14,15,16,17,18]. The role of micropores in Fe-N-C catalysts seems to be quite clear. However, the role of meso-/macropores is not so clear [13,19,20]. It is believed that mesopores enhance electrolyte wetting, increasing the electrochemically available active sites, while macropores improve mass transport by acting as conduits, ensuring the efficient delivery of reactants to the active sites and a good contact between the ionomer and the active sites [17]. However, a consensus establishing a direct relationship between the porous structure and catalytic activity has not yet been reached [13,19,21]. To investigate how the porous structure affects catalyst activity, synthetic matrices with easily adjustable porosity, such as carbon gels, can be utilized [22].

Carbon gels have been reported as potential materials for a broad range of uses, including as supercapacitors [23,24], adsorbents for the removal of contaminants [25,26,27], hydrogen storage [28], thermal insulators [29,30], and carbon supports for electrocatalysts (e.g., oxygen reduction reaction) [31,32,33]. Carbon gels are categorized into various types based on their drying method. Among these types, carbon xerogels (CXG) utilize the most cost-effective drying method, aligning with our goal of reducing catalytic layer costs [34,35]. CXG offer a broad range of tunable texture properties, allowing for the precise control of their porous structure by adjusting synthesis variables, such as the sol-gel solution’s pH or the reactants’ ratio [36,37,38]. Studies have shown that the pH of the polymerization process mainly affects the inter-particle voids, leading to the formation of wider pores (meso- and macropores), while micropores’ volume remains relatively unaffected [39].

Using CXG as a matrix in the preparation of Fe-N-C catalysts avoids the need for templates, such as those employed in the sacrificial support method (SSM) [36,37]. This method allows for the accurate control of catalyst porosity by varying the size of silica precursor. Other catalysts are synthesized by hard-templating, with the aim of optimizing the porous structure [40,41]. However, the use of templates presents processing and environmental challenges [42,43].

In a previous work [44], we synthesized Fe-N-C catalysts through a one-step template-free approach using CXG as a matrix, proving the feasibility of this carbonaceous structure as a matrix for iron- and nitrogen-based catalysts. To achieve this, iron and nitrogen precursors were mixed with an organic xerogel (prior to carbonization) and treated at 1050 °C for 1 h, leading to N and Fe doping and formation of a porous structure simultaneously. In the present work, we suggest the use of carbon xerogel (instead of the organic type) for mixing with iron and nitrogen, with the aim of assessing the role of the porosity of the carbon material in catalytic activity. The objective of this research is to study the influence of the porous structure of the matrix on the catalytic activity of Fe-N-C against ORR in acidic media. For this purpose, CXG synthesized at different pH values, with a large range of pore sizes, have been doped with Fe and N using a template-free method. In contrast to our previous work [44], the CXG synthesis process preserves the porous structure of the carbon material, enabling the elucidation of the impact of macro- and mesopores on the catalytic activity of Fe-N-C. In addition, the one-stage synthesis method of our previous work [44] has been compared with the two-stage synthesis method of this work in order to assess if different active sites are created and the microporous structure’s role. It is important to note that the findings derived from ideal three-electrode systems may not directly translate to real fuel cell devices. Therefore, the activity and stability of the catalysts will also be evaluated in a single-cell fuel cell configuration. This approach serves to demonstrate the crucial role played by the porosity and structure of the carbon matrix alongside the synthesis method in the performance of Fe-N-C catalysts for ORR in a proton exchange membrane fuel cell (PEMFC).

## 2. Experimental

### 2.1. Carbon Xerogels Synthesis

In this work, a sol-gel approach has been applied for the synthesis of carbon xerogels (CXG) at different pH values. This method is based on the polycondensation of formaldehyde (F) and resorcinol (R) [45,46]. The reaction was performed in an aqueous medium with the use of NaOH as a base agent. Resorcinol was first dissolved in deionized water, and the pH was set to 5.5 through the addition of a few drops of diluted NaOH (0.5 M NaOH). The next step was the addition of formaldehyde and the stirring of the mixture until a homogeneous solution was obtained. Two key parameters in the synthesis of CXG, R/F ratio and dilution (D), were kept constant at 0.5 and 5.7, respectively. Afterward, through the addition of NaOH or nitric acid (0.5 M HNO_3_), the pH was adjusted to the target value (4.5, 5.8, 6, 7, 8). The obtained solutions were poured into glass vials and stored in an oven for 24 h at room temperature, 24 h at 50 °C, and 72 h at 85 °C for curing and gelation [47,48]. In order to preserve their porous structure, the resulting gels were embedded in ethanol for 72 h, and this ethanol was exchanged three times every 24 h. The aqueous gels were then dried at 65 °C for 5 h and at 110 °C for another 5 h [31,49]. Finally, pyrolysis of the gels was performed under an inert atmosphere (N_2_) at 800 °C in a tubular oven for 3 h. The carbon xerogels obtained were named CXG-*n*, where *n* corresponds to the pH values used for organic xerogel synthesis.

### 2.2. Fe-N-CXG Synthesis

Iron acetate and urea were the precursors of iron and nitrogen, respectively. Two solutions were initially prepared. In the first one, 400 mg of urea and 63 mg of iron acetate (FeAc, 95% Sigma-Aldrich, St. Louis, MO, USA) were dissolved in 10 mL of a mixture comprising a blend of deionized water and ethanol (96% analytical grade, Labkem, Barcelona, Spain). In the second, the previously prepared CXG-*n* (2 g) was dispersed via sonication in 30 mL of deionized water. The solution containing the urea and FeAc and the carbon-containing solution were then mixed and magnetically stirred for 1 h. The powder was ground in a planetary ball mill for three hours, following the procedure described in previous works [44,50]. The ball to powder ratio was 40:1 [51,52]. The resulting powder was pyrolyzed at 1050 °C under a N_2_ atmosphere for 1 h followed by quenching (fast cooling) [53]. The catalysts were labelled as Fe-N-CXG-*n*, where *n* is the pH value used during the carbon material’s polymerization. To study the involvement of nitrogen in these catalysts a nitrogen-free catalyst was also synthesized. The same procedure described was followed but without using urea. Iron acetate was mixed with CXG-6 obtaining the catalyst labelled as Fe-CXG-6.

Finally, to remove the non-active iron particles and maximize catalytic activity, the catalysts were subjected to three cycles of acid leaching followed by a thermal treatment [54,55]. The acid leaching was carried out in 0.1 M HClO_4_ for 15 min at 60 °C, and the thermal treatment took place in a tubular reactor at 950 °C for one hour in an inert atmosphere [19].

### 2.3. Physical-Chemical Characterization

The porosity of the materials was investigated using nitrogen physisorption, Hg porosimetry, and helium picnometry. Nitrogen adsorption/desorption isotherms were performed at −196 °C in an ASAP 2020 from Micromeritics (Norcross, GA, USA). The total surface area (S_BET_) was calculated using the Brunauer–Emmer–Teller equation. The surface area corresponding to the micropores (S_micropore_) was calculated using the t-plot method. The total pore volume (V_pore_) was determined at a relative pressure of P/P^0^ = 0.99. For the micropore volume (V_micro_), the Dubinin–Astakhov method was used. V_mesopore_ was evaluated following the methodology described in reference [56]. Hg porosimetry and helium pycnometry were performed with AUTOPORE V and ACCUPYC II, respectively, both belonging to Micromeritics. For the macroporous samples, the total pore volume (V_v_) was calculated using the following equation:

V_v_ = V_DUB_ + V_Hg_ (1)

The mercury porosimetry technique is inadequate for determining pores narrower than 7.5 nm, and nitrogen physisorption does not determine pores wider than 50 nm. Therefore, by neglecting pores between 2 and 7.5 nm, both techniques can be combined for V_v_ calculations [57]. The results section describes how this approach was applied to the samples in this paper.

The real density was obtained using helium picnometry. The porosity percentage was calculated using the V_v_ and the real volume (V_real_, inverse of real density) using the following equation:

(2) $$\%\;\mathrm{porosity}=\frac{V_{v}}{V_{v}+V_{\mathrm{real}}}$$

Scanning electron microscopy (SEM) evaluated the morphology of the samples on a SEM microscope (Hitachi 3400 N Tokyo, Japan). The chemical composition was assessed using elemental analysis (EA), atomic emission spectrometry with inductive coupling plasma (ICP-AES), and X-ray photoelectron spectroscopy (XPS). The EA used a Thermo Flash 1112 analyzer, while the ICP-AES a Xpectroblue-EOP-TI FMT26 (Spectro, Kleve, Germany). XPS was analyzed in an ESCA Plus Omicron spectrometer with a 225 W (15 mA, 15 kV) power and Al (1486.7 eV) anode. The CasaXPS 2.3.15 software was used to process the results and for the deconvolution of the N1s spectra, considering the Shirley background and shape of the peaks, 70% Lorentzian and 30% Gaussian.

### 2.4. Electrochemical Characterization

The performance of the Fe-N-CXG-*n* catalysts against the ORR was determined using a microAutolab potentiostat/galvanostat (Metrohm, Herisau, Switzerland) in a three-electrode setup at room temperature. A rotating disk electrode (RDE) was used as the working electrode. The RDE consists of a glassy carbon disc with a diameter of 5 mm. The reference electrode employed was a reversible hydrogen electrode (RHE), while a high surface area glassy carbon rod served as the counter electrode. An ink was prepared with the catalyst and deposited, using the drop-casting method, on the glassy carbon disc. The ink was formulated by combining 300 µL of a solution of isopropanol and water (1:3 vol) with 7 mg of the investigated catalyst and a Nafion^®^ perfluorinated resin dispersion. To achieve 15 wt% of the total catalyst layer, 15 μL of 10 wt.% Nafion^®^ was used. The loading of the Fe-N-CXG-*n* catalyst was 600 μg cm^−2^. The activity of Pt versus ORR has also been tested for comparative purposes. For this purpose, a commercial 40 wt.% Pt/C catalyst (HiSPEC 4000, Alfa Aesar, Tewksbury, MA, USA) was used with a loading of 50 μg_Pt_ cm^−2^. All the measurements pertaining to the ORR were performed in a 0.5 M H_2_SO_4_ electrolyte solution saturated with oxygen, and the scan rate for the linear sweep voltammograms was set to 2 mV s^−1^.

To determine the quantity of electrons transferred, the polarization curves were recorded at different electrode rotation speeds (400, 600, 900, 1600, and 2500 rpm) by applying the Koutecky–Levich method, as in previous works [44,50].

### 2.5. Fuel Cell Tests

The cathodic performance of the Fe-N-C catalysts was evaluated in a single-cell configuration of a polymer electrolyte fuel cell. To prepare the electrodes, an ink solution was created by dispersing the Nafion^®^ and the catalyst in isopropyl alcohol, maintaining an ionomer-to-catalyst ratio of 0.82. After subjecting the ink solution to sonication for 20 min, it was sprayed onto carbon paper (Sigracet GDL-39BC, Ion Power, Munich, Germany) to form the electrode. The catalyst loading in the electrode was set to 4 mg cm^−2^. A similar procedure was followed to prepare the anode, utilizing a commercial Pt/C catalyst (40 wt.% Pt, HiSpec 4000, Alfa Aesar) with 33 wt.% of Nafion^®^ and a Pt loading of 0.2 mg cm^−2^. The assembly of the membrane–electrode assemblies (MEAs) involved hot-pressing the cathode and anode with a pre-treated Nafion^®^ NR212 membrane (140 µm, Ion Power) at 125 °C and a pressure of 25 kgf cm^−2^ for 5 min. Nafion-based membranes are commonly used in fuel cells [20,58,59,60,61,62,63,64].

The MEA was placed in a single cell. The single cell had serpentine flow channels on both sides and was 5 cm^2^ in size. The cell temperature was measured close to the cathode side and near the flow channel and was maintained at 80 °C using external heating. The fuel cell experiments were conducted using a Fuel Cell Technologies Inc. station (Albuquerque, NM, USA). The cell was supplied with pre-heated (85 °C) and fully humidified oxygen and hydrogen. The flow rates at the cathode and anode were adjusted to 1.5 and 1.3 times the stoichiometric values, respectively, while maintaining backpressure at 150 kPa for the cathode and 130 kPa for the anode. Prior to the experiments, the MEA underwent a conditioning process consisting of five constant voltage steps, each lasting 5 min and varying the voltage between 0.8 and 0.4 V. These operating conditions comply with the standardized fuel cell testing protocols for single-cell configuration in automotive applications [65].

## 3. Results and Discussion

### 3.1. Physicochemical Characterization of CXG

**Figure 1:**
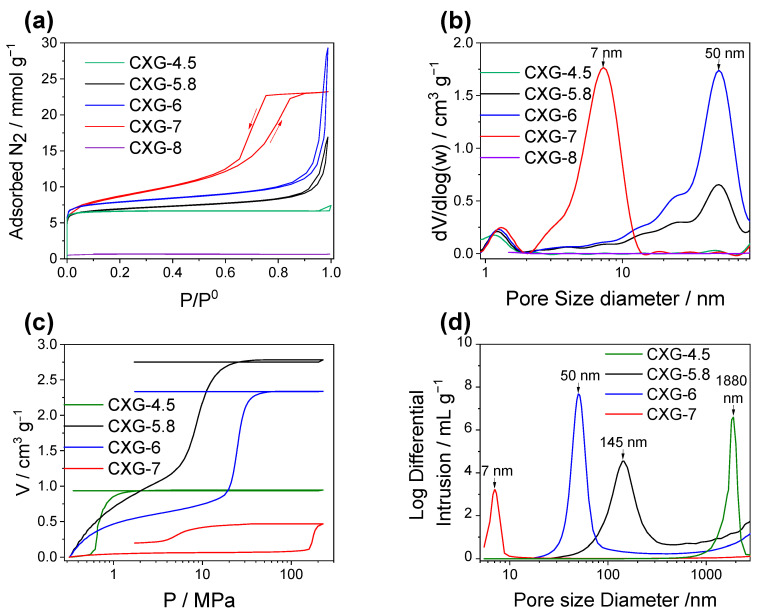
(**a**) Nitrogen physisorption isotherms and (**b**) pore size distribution obtained from N_2_ adsorption/desorption isotherms; (**c**) Mercury porosimetry curves; (**d**) pore size distribution obtained from mercury porosimetry.

First, the porous structure of the carbon xerogels used as a matrix in the catalysts is studied. Figure 1a and 1b represent the isotherms and pore size distribution, respectively, obtained by nitrogen physisorption of the synthesized xerogels at pHs = 4.5, 5.8, 6, 7, and 8. The variation of the isotherm shape shows how the synthesis pH has a significant influence on the mesoporous pore structure of the xerogel [66,67]. The xerogels synthesized at a low pH (CXG-4.5) present an I-type isotherm according to the IUPAC classifications, which is related to microporous materials without mesoporosity. As the pH increases, the isotherm shape varies: CXG synthesized at pHs of 5.8 and 6 present combined type I and II isotherms, whereas CXG-7 presents a type IV isotherm with hysteresis, and, for a pH higher than 7, the material becomes totally non-porous (CXG-8). The pore size distribution also changes in the mesopore zone with the pH (Figure 1b). All the CXG samples, except for CXG-8, exhibit a peak at 1.2–1.3 nm associated with the micropores. Furthermore, the CXGs prepared at pHs of 5.8 and 6 show a clear peak around 50 nm indicating the presence of meso- and macropores alongside some contribution of mesopores around 12 and 23 nm. CXG-7 shows a clear peak at 7 nm, demonstrating the presence of smaller mesopores.

Figure 1c shows the mercury porosimetry intrusion/extrusion curves of the xerogels synthesized at pHs = 4.5, 5.8, 6, and 7. Sample CXG-7 is compressed without any intrusion of mercury inside the pores, until it reaches high-pressure values, which confirms that CXG does not present macropores and that the size of the mesopores is small. The Washburn equation was applied to calculate the pore size distributions (Figure 1d). Monomodal pore size distribution is obtained, which is characteristic of carbon gels [68]. In the samples synthesized below a pH of 7, mercury penetrates at a lower pressure, which indicates the presence of larger mesopores and macropores [38,69]. The pore size distribution calculated from Hg porosimetry coincides with that of N_2_ physisorption and corroborates that, with increasing pH, the size of the macro-mesopores decreases. However, as shown in Figure 1b from the nitrogen physisorption, the size of the micropores remains constant.

Table 1 shows all the data obtained from the nitrogen physisorption, mercury porosimetry, and He pycnometry techniques. The values calculated from nitrogen physisorption (surface area, pore volume, and micropore volume) increase with increasing values of pH until a pH of 7 is exceeded, at which point the structure of the carbon material collapses, and the surface area is significantly decreased. In the present work, above a pH of 7, the structure of the CXG collapses, with this pH value being higher than the pH value at which collapse occurs in other studies reported in the literature (around pH 6–6.5) [66,70]. This indicates that the synthesis conditions followed in this work (solvent exchange and sub-critical drying) avoid collapse in syntheses at higher pHs, thus obtaining CXG with higher S_BET_ values (696 m^2^ g^−1^ for CXG-7) than those reported in the literature [71,72,73,74]. Although CXG-7 presents the highest surface area values, its pore volume values are lower, due to its narrower pores. Taking into consideration the values provided using mercury porosimetry (considering the contribution to porosity of the macropores), CXG-5.8 has the highest value for total volume and porosity percentage (3.04 cm^3^ g^−1^ and 85%, respectively). The pore size calculated from both nitrogen physisorption and mercury porosimetry is also reported in Table 1. The V_v_ of the CXGs synthesized without collapse (excluding CXG-8) varies from 0.8 to 3.04 cm^3^ g^−1^. The V_v_ is calculated using Equation (1) for CXG synthesized at pHs = 4.5, 5.8, and 6, given that their pore volume between 2 and 7.5 nm cm^3^ g^−1^ can be considered negligible. In the case of CXG synthesized at pH = 7, the V_v_ corresponds to the V_pore_ calculated using nitrogen physisorption, as macropores are not present. Moreover, the mesopore and macropore diameters of the CXGs synthesized in this work are quite wide, ranging from 7 to 1880 nm.

The results summarized in Table 1 demonstrate the easiness of synthesizing CXG with a tailor-made porous structure by modifying only one synthesis variable, the pH value of the R-F solution.

**Figure 2:**
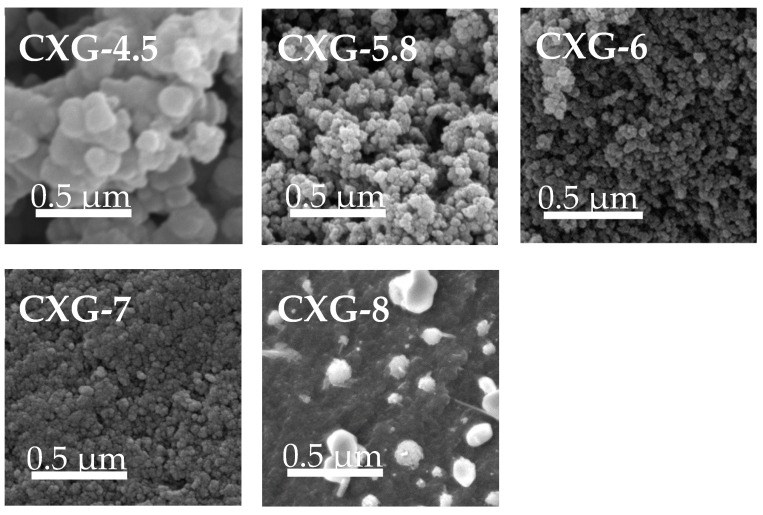
SEM micrographs at a 50,000 magnification.

The SEM images of the CXGs studied in the present work are shown in Figure 2. As is often found in carbon gels [70,75], the images represent materials made up of agglomerates of interconnected spherical particles forming well-opened accessible macropores and/or mesopores. While the micropores are located inside the primary particles (not visible with SEM), the meso- and macropores develop between the particles when the solvent is removed [76]. The SEM results agree with the porosity analyses previously mentioned. At a pH of 4.5, the particles are larger (see Figure S1 in the Supplementary Materials), and, therefore, the channels forming the macropores are wider with an average size of 1880 nm, according to the porosimetry analysis. As we increase the synthesis pH (up to 7), the size of the spheres is reduced (Figure S1), giving rise to more compact structures with narrower meso-macropores. When the pH goes even higher, as is the case for CXG-8, the structure becomes much more compact because of the collapse of the structure, and, moreover, salt deposits from the NaOH residues that have not reacted in the formation of the gel are observed (bright points in the image).

Pekala et al. [77,78] established that the structure of the xerogels is a consequence of the competition between two reactions: the addition and condensation reactions. In the addition reaction, formaldehyde is added to the resorcinol ring, giving rise to the monomers necessary for polymerization. Simultaneously, in the condensation reaction, cations are formed from the monomers [79]. These cations react with other RF monomers through methylene groups (-CH_2_-) and methylene–ether bridges (-CH_2_OCH_2_-) [77], forming aggregates. These aggregates continue to grow and crosslink each other to build up the solid final gel form [37]. As can be deduced, the pH at which the polymerization process takes place affects the development of both reactions: the addition reaction is favored at high pH values, while the condensation reaction is favored at low pH values. Therefore, at higher pHs, more clusters are formed because the addition reaction predominates. These clusters are smaller, creating narrower meso-macropores. At lower pHs, the opposite happens, i.e., condensation is favored [37] and fewer but larger clusters are formed, resulting in larger pores.

In summary, the CXGs synthesized in this work present different types of porous structures. By simply varying the synthesis pH, a broad range of carbon materials with different porous properties can be obtained: one micro-/macroporous material (CXG-4.5), one material with a combination of micro-, meso-, and macroporosity (CXG-5.8), two micro-/mesoporous materials (CXG-6 and CXG-7), and one non-porous material (CXG-8).

### 3.2. Physicochemical Characterization of Fe-N-CXG

In this section, the physicochemical characterization of Fe-N-CXG catalysts is investigated. The chemical composition calculated via EA and ICP is shown in Table 2. The percentage of Fe and N remains constant in the different catalysts, showing that the porosity of the carbonaceous matrix does not modify the chemical composition of the Fe-N-C catalysts. Further information about the composition of the Fe-N-C catalysts can be found in Table S1.

**Figure 3:**
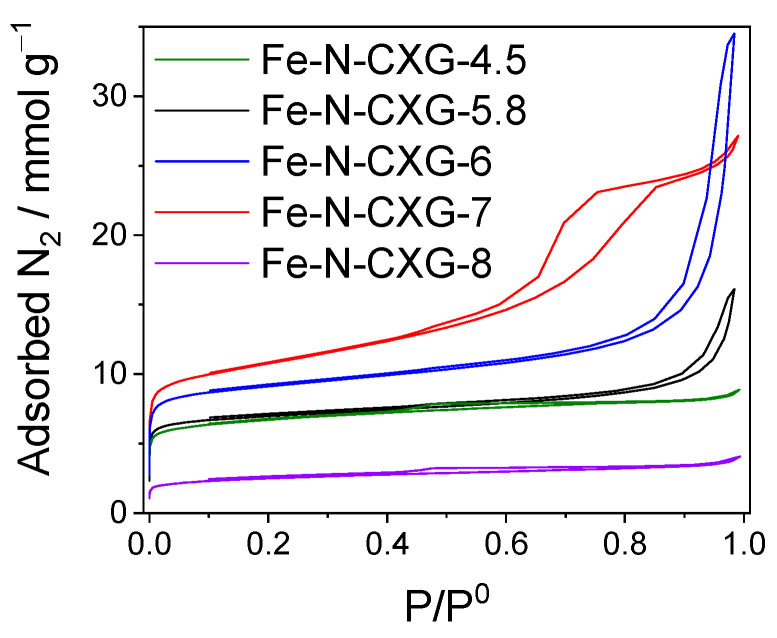
Nitrogen physisorption isotherms for Fe-N-CXG-*n* catalysts (CXGs obtained at different pHs).

The results of the Fe-N-CXG catalysts subjected to three cycles of acid leaching and thermal treatments will be presented hereafter. The textural properties of the Fe-N-CXG-X were also assessed using N_2_ physisorption (Figure 3). The porous structure of the CXGs is mostly preserved after N and Fe doping. In fact, comparing Figure 3 with Figure 1a, the same trend is observed. In the supporting data (Figure S2 and Table S2), the variation in the porous structure of the materials in the different stages of the doping process is explained.

Table 3 summarizes the data extracted from the N_2_ physisorption of the catalysts synthesized at different pHs. It is shown that Fe-N-CXG-7 presents the highest surface area value (891 m^2^ g^−1^), while the catalyst synthesized with a CXG at a pH of 6 exhibits the highest volume of mesopores (0.94 cm^3^ g^−1^).

### 3.3. Activity towards the Oxygen Reduction Reaction in an Acidic Medium

**Figure 4:**
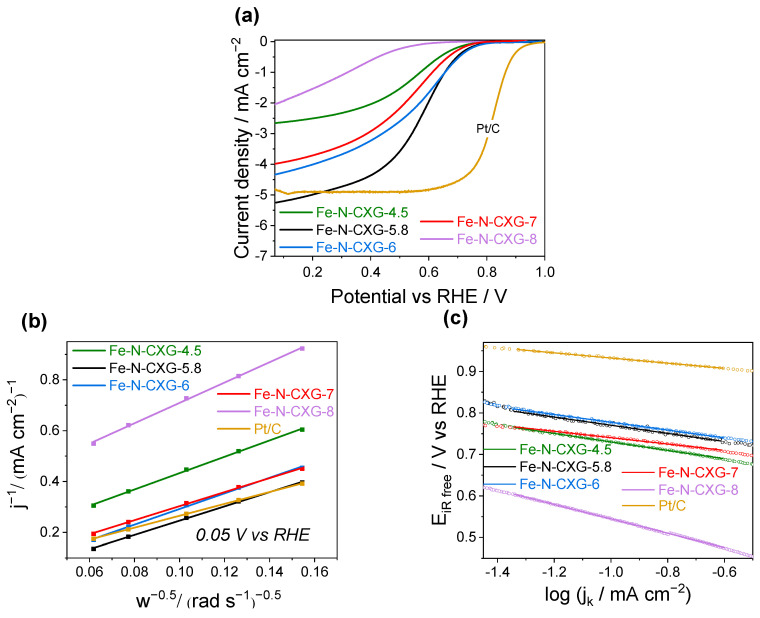
(**a**) LSVs in RDE at 1600 rpm. (**b**) Koutecky–Levich plot calculated at 0.05 V vs. RHE. (**c**) Tafel diagram from LSV at 1600 rpm for Fe-N-CXG-*n* and Pt/C catalysts against the ORR in an O_2_-saturated 0.5 M H_2_SO_4_ electrolyte.

The performance of the catalysts prepared in this work was evaluated towards the ORR in an RDE in a 0.5 M H_2_SO_4_ aqueous electrolyte using linear sweep voltammetry (LSV), the Koutecky–Levich (K-L) method, and the Tafel method. The effect of the porous structure of a catalyst’s matrix on the ORR activity is examined (Figure 4). The electrochemical values (E_onset_, E_1/2_, j_d_, n, and Tafel slope) calculated from Figure 4 are collected in Table 4. Here, the activity of the catalysts subjected to three cycles of AL/TT is presented. For further information on the effect of nitrogen and iron alongside the effect of the acid leaching/thermal treatments on performance, see the Supporting Information (Figures S3–S5 and Tables S3 and S4).

The electrocatalytic activity demonstrates an increase as the synthesis pH of the CXG matrices is raised from 4.5 to 5.8, with the Fe-N-CXG-5.8 catalyst being the one with the best activity against ORR in acidic media. This fact emphasizes the important role of macropores in favoring the mass transport of reactants to the available active sites located in the micropores [9]. Wang et al. [1] also employed CXG as matrices for Fe-N-C materials, and it was reported that catalysts with larger pore diameters yielded higher diffusion-limited current densities. Consistent with this finding, in this paper, the catalysts prepared at higher pH (pHs = 6 and 7) values (featuring narrower meso-macropores) exhibit lower j_d_ values. The presence of the mesopores, according to Shui et al. [80], creates additional tortuosity between the reactants and the catalytic site located in the micropores. Therefore, resistance to mass transfer is increased. Furthermore, the volume/surface area ratio is higher in mesopores than in micropores. This feature adds a considerable volume to the catalyst and reduces the volumetric current density of the electrode.

**Figure 5:**
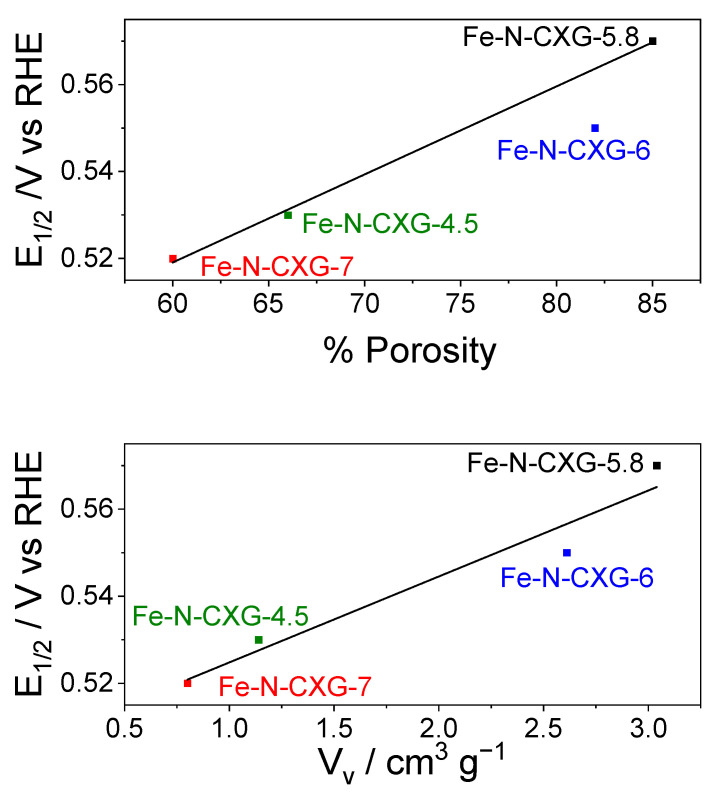
Correlation between the half-wave potential and the porosity (**above**) and the total pore volume V_v_ (**below**).

In addition, Figure 5 shows the correlation between E_½_ and the total pore volume (above) and the porosity percentage of the carbonaceous matrix used (in this case, the CXG synthesized at different pHs; below). This study supports the importance of pore structure in the activity of Fe-N-C catalysts towards ORR in acidic media.

In previous research, we obtained Fe-N-CXG catalysts using a template-free method in a single step: iron and nitrogen were mixed with an organic xerogel (before carbonization) and treated at 1050 °C, for 1 h [44]. In the present work, the use of a carbon xerogel (instead of the organic one) mixed with iron and nitrogen is proposed in a two-step synthesis, with the aim of assessing if catalytic activity improves as a consequence of using the organic or carbonized materials. Therefore, here, we compare a catalyst from our previous work using an organic xerogel as the carbon precursor, obtained at a pH of 5.8 (here named as Fe-N-OXG-5.8), with the Fe-N-CXG-5.8 catalyst obtained in this work.

**Figure 6:**
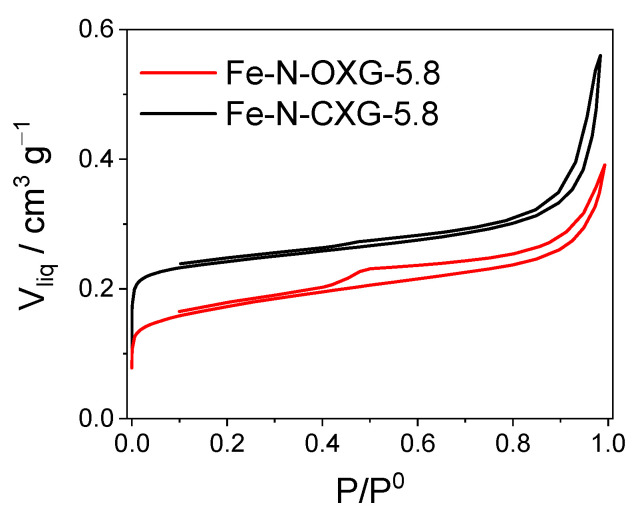
Nitrogen physisorption isotherms for the catalyst obtained in a single-stage synthesis process (Fe-N-OXG-5.8), in our previous work [44], with respect to the one obtained using the two-stage synthesis method from this work (Fe-N-CXG-5.8).

First, the porous structure of both materials has been compared. Figure 6 shows the adsorption–desorption isotherms calculated via nitrogen physisorption, and Table 5 shows the data obtained.

Fe-N-CXG-5.8 shows a higher microporosity development than Fe-N-OXG-5.8, probably due to the slower pyrolysis process. The micropore volume and micropore area increase from 0.1 to 0.23 cm^3^ g^−1^ and from 334 to 519 m^2^ g^−1^, respectively, when introducing an additional step in the synthesis process. It is worth noting the micropores’ key role in Fe-N-C catalysts, as it has been proven that Fe-N active sites are hosted in micropores [15,16,17,18,81].

As mentioned above, the porous properties of CXG are easily tailored through small changes in the synthesis process. While macro- and mesoporosity are modified in the polymerization step by varying the synthesis pH, microporosity can be tuned in the pyrolysis step [82,83]. In the single-stage process following the procedure outlined in our previous work [44], OXG undergoes carbonization simultaneously with Fe and N doping. This results in a fast carbonization, with a short time to form micropores, also entailing the collapse of the structure because of the fast evaporation of volatile species. However, in the case of the catalyst synthesized in two stages, the porous structure is formed before doping with Fe and N, since a previously carbonized CXG is employed. The heating rate during the carbonization process applied to CXG before doping with Fe and N to form Fe-N-CXG-5.8 is much slower, allowing for the full formation of micropores, also avoiding pore collapse. These micropores are retained after the doping procedure, as depicted in Figure S2.

Table 6 shows the chemical composition determined from an elemental analysis of the catalysts synthesized with OXG (Fe-N-OXG-5.8) and CXG (Fe-N-CXG-5.8). The amount of N introduced following the one-step procedure, Fe-N-OXG-5.8, is slightly higher (0.72 wt.%) than that introduced in the two-step procedure, Fe-N-CXG-5.8, (0.6 wt.%), although the N/C ratio is equal in both samples (0.007).

In addition to the amount of N introduced, it is also valuable to compare the nitrogen functionalities, which were determined by means of XPS. Figure S6 shows the deconvolution of the N1s orbital signal into five peaks: N-pyridinic (398.2–398.7 eV), metal-bound nitrogen, N_x_-M (399.5–399.8 eV), N-pyrrolic/pyridonic (400.5–400.9 eV), N-graphitic/quaternary (401.5–402 eV), and N-oxide (402.5–403 eV). The peak associated with nitrogen bonded to metal (iron in this case) is indicative of the number of active sites [84,85]. Table 7 provides the chemical speciation for both catalysts. It is noteworthy that Fe-N-CXG-5.8 shows a higher prevalence of N_x_-M species (17.2%) compared to Fe-N-OXG-5.8 (12.1%). This can be attributed to the fact that, as we have previously observed, Fe-N-CXG-5.8 features a greater quantity of micropores (Figure 6 and Table 5), and microporosity plays an essential role in the formation of iron–nitrogen centers [59]. It is known that N_x_-M moieties are related to the reduction of O_2_ to H_2_O directly (4 e^−^) [86,87], which could favor its catalytic activity.

Other important N moieties in Fe-N-C catalysts are N-pyrrolic and N-pyridinic due to their contributions to the ORR mechanism. N-pyrrolic corresponds to nitrogen atoms incorporated in five-membered heterocyclic aromatic rings and participates in the partial reduction of O_2_ to H_2_O_2_, while N-pyridinic is incorporated in a six-membered ring [88] and acts in the reduction of H_2_O_2_ to H_2_O [87,89,90,91,92]. Hence, to ensure a high conversion of O_2_ to H_2_O, it is advantageous to maintain a high N-Pyridinic/N-Pyrrolic ratio. Notably, Fe-N-CXG-5.8 presents elevated N-Pyridinic percentages and reduced N-Pyrrolic percentages compared to Fe-N-OXG-5.8, resulting in a superior N-Pyridinic/N-Pyrrolic ratio.

**Figure 7:**
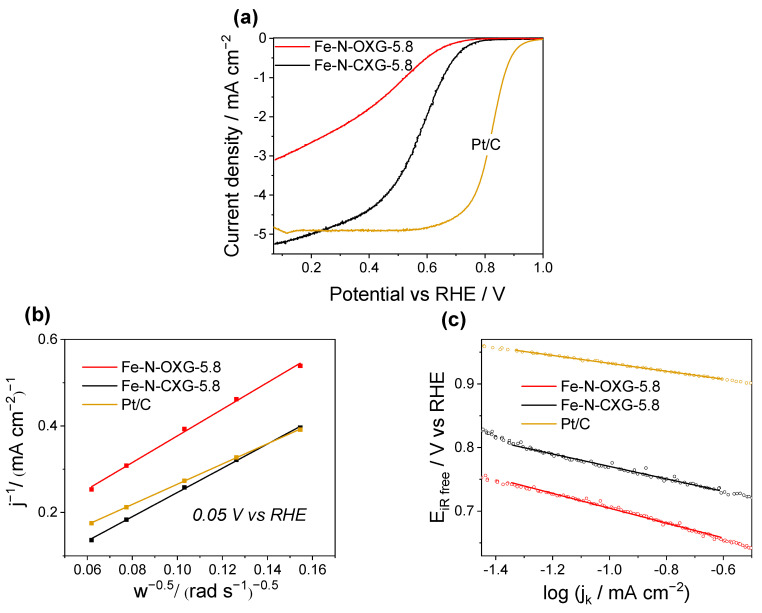
(**a**) LSVs in RDE at 1600 rpm. (**b**) Koutecky–Levich plot calculated at 0.05 V vs. RHE. (**c**) Tafel diagram from LSV at 1600 rpm for Fe-N-OXG-5.8, Fe-N-CXG-5.8, and Pt/C catalysts against the ORR in an O_2_-saturated 0.5 M H_2_SO_4_ electrolyte.

The electrocatalytic activity of Fe-N-OXG-5.8 and Fe-N-CXG-5.8 against ORR is compared below. Figure 7a shows the linear sweep voltammetry (LSV) polarization curves at 1600 rpm, in an oxygen-saturated 0.5 M H_2_SO_4_ solution. The reaction starts at a higher potential (E_onset_, Table 8) for catalyst Fe-N-CXG-5.8 (two-step synthesis). In addition, this catalyst exhibits a higher half-wave potential (E_1/2_ = 0.44 compared to 0.57 V vs. RHE) and a superior limiting diffusion current (j_d_ = −3.1 compared to −5.2 mA cm^−2^). These values indicate that the two-step synthesis method produces catalysts with enhanced performance against ORR in an acidic medium compared to the catalysts synthesized in a single step. As mentioned above, the catalysts synthesized in two stages present a larger microporosity, facilitating the creation of a larger number of Fe-N active sites.

The reaction mechanism has been investigated using the Koutecky–Levich method. Figure 7b plots the inverse of the current density against the inverse square root of the rotation speeds (400, 600, 900, 1600, and 2500 rpm, shown in Figure S7) at 0.05 V vs. RHE. The number of electrons exchanged in the reaction (n) is calculated from Figure 7b [93]. Fe-N-CXG-5.8 shows a higher number of exchanged electrons (n = 3.4) than Fe-N-OXG-5.8 (n = 3.1). As previously indicated (Table 7), Fe-N-CXG-5.8 exhibits a higher percentage of N_x_-M groups and a greater N-Pyridinic/N-Pyrrolic ratio. Both parameters promote the complete conversion of O_2_ to H_2_O following 2 × 2 e^−^ or 4 e^−^ mechanisms. The Tafel slope is calculated in Figure 7c, and the values are enumerated in Table 8. The Tafel slope of Fe-N-CXG-5.8 is lower (98 mV dec^−1^) than the Tafel slope of Fe-N-OXG-5.8 (117 mV dec^−1^), indicating that the reaction catalyzed by Fe-N-CXG-5.8 is kinetically faster and, therefore, more efficient.

In addition to the three-electrode cell, it is necessary to measure the activity of a catalyst in a full device to determine its practical feasibility. Fe-N-OXG-5.8 and Fe-N-CXG-5.8 have been employed as cathodic catalysts in an H_2_–O_2_ single fuel cell equipped with a Nafion^®^ membrane. In this work, the catalyst-coated substrate (CCS) procedure has been applied for the fabrication of the MEAs. Firstly, the catalyst is sprayed onto the porous carbon paper to prepare the GDE and is then hot-pressed together with the membrane [94,95]. In laboratory studies, the CCS method is commonly utilized owing to its easy operation and control [96]. However, for industrial production, it is preferable to use the catalyst-coated membrane (CCM) method for the preparation of MEAs, since CCS offers a low efficiency. In the CCM method, the membrane is coated with the catalyst directly and then pressed with the gas diffusion layer (GDL) [97].

**Figure 8:**
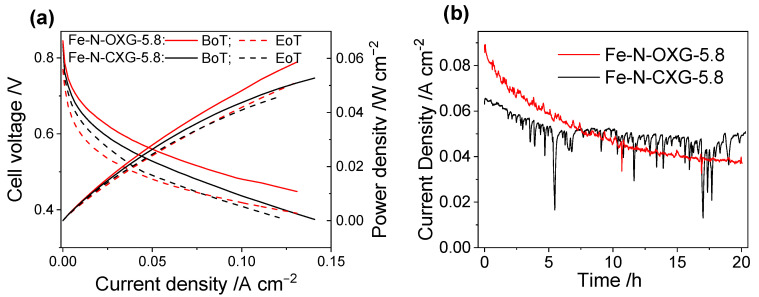
(**a**) Polarization curves corresponding to MEAs, including cathodes made with Fe-N-CXG-5.8 and Fe-N-OXG-5.8 catalysts (4 mg cm^−2^) and anodes made with 40%Pt/C (0.2 mgPt cm^−2^) and a Nafion^®^ NR212 membrane; BoT and EoT of the durability tests (potential holding at 0.5 V for 20 h). Operating conditions: 80 °C; H_2_/O_2_ at λ = 1.3/1.5; 100% RH; and back pressure of 1.5 bar-gauge. (**b**) Current density recorded during the durability tests (potential holding at 0.5 V for 20 h).

On the other hand, despite significant progress in the development of Fe-N-C catalysts, their stability remains a critical issue [98,99,100]. Several degradation mechanisms have been suggested for Fe-N-C catalysts, including metal loss and corrosion of carbon and nitrogen components [101], protonation leading to anion adsorption [102], hydroperoxyl radical attack [103], and micropore blockage due to water accumulation [104]. Therefore, the stability of Fe-N-OXG-5.8 and Fe-N-CXG-5.8 catalysts has also been assessed through a durability test that consisted in keeping the fuel cell operating at 0.5 V for 20 h. Figure 8a illustrates the polarization curves at the beginning (BoT) and at the end (EoT) of the durability test. The current density produced during the 20 h at 0.5 V is represented in Figure 8b.

The polarization curves at the BoT and EoT of the durability test showed that, surprisingly, in contrast to the results obtained from the three-electrode cell, Fe-N-OXG-5.8 displayed comparable activity to Fe-N-CXG-5.8 in the PEMFC. This underscores the importance of testing catalysts in a real fuel cell device because the information obtained from electrochemical characterization in RDE experiments only provides partial insights into a catalyst’s behavior, particularly its intrinsic activity. In PEMFC, other factors can come into play, such as water accumulation in the electrodes. In fact, water management in fuel cells is a critical issue [105]. If the mass transfer process is slowed down, water is not properly evacuated [106,107]. For the Fe-N-CXG-5.8 catalyst, current density peaks are observed (Figure 8b), which are likely associated with water accumulation on the electrode’s surface. The peaks are more frequent and intense in the Fe-N-CXG-5.8 catalyst due to its more microporous structure, impairing mass transfer. This water accumulation can block the access of reactants to the micropores [79], thereby reducing the catalyst’s performance.

Regarding durability, notable differences are observed between the catalyst synthesized in a single step, Fe-N-OXG-5.8, and the catalyst synthesized in two steps, Fe-N-CXG-5.8. Upon the durability test, Fe-N-OXG-5.8 showed a current density loss of 45%, whereas Fe-N-CXG-5.8 showed a loss of only 22%. This variation can be attributed to the difference in the formation of the catalyst matrix’s porous structure. As explained above, the CXG acting as the matrix in catalyst Fe-N-CXG-5.8 is formed through a slow and controlled carbonization process, whereas, for Fe-N-OXG-5.8, carbonization is much faster, potentially resulting in a more fragile and easily degradable structure. The degradation percentages (45% and 22%) are consistent with those obtained in a previous work dealing with Fe-N-C catalysts using in situ nitrogen-doped carbon xerogels [50]. It is challenging to compare these data with the rest of the literature as different degradation procedures are followed [61]. To further illustrate this point, Kiciński et al. investigated the stability of Fe-N carbon gel catalysts over 50 h at 0.5 V [20]. The findings from this investigation indicate that a substantial decline in activity takes place during the initial operational hours, resulting in a current density drop of 60% after 24 h of continuous operation.

## 4. Conclusions

CXG are materials with a porous structure that can be easily adjusted by modifying synthesis properties, such as the pH. The utilization of CXG as matrices for Fe-N-C catalysts allows researchers to study how the porous structure influences the electrocatalytic performance of Fe-N-C catalysts against ORR in acidic media. The catalyst using CXG synthesized at a pH of 5.8 as the matrix (Fe-N-CXG-5.8) shows the highest activity for the ORR compared to the other catalysts. This is attributed to its structure, which comprises micropores, macropores, and a minor presence of wide mesopores. The macropores favor the mass transport of reactants, while the narrower mesopores may introduce additional tortuosity between the reactants and the catalytic site located in the micropores.

Finally, by comparing two catalysts synthesized with the same matrix (CXG-5.8) but following a different doping procedure, it is concluded that the presence of micropores is critical to the formation of Fe-N active sites. The catalyst synthesized in two steps (Fe-N-CXG-5.8) preserves the porous structure of the CXG better, showing more micropores than the catalyst synthesized in one step, Fe-N-OXG-5.8. A higher presence of micropores leads to the formation of more N_x_-M species and enhances catalytic activity in a three-electrode cell. In contrast, an increased presence of micropores results in water accumulation during catalyst testing in a PEMFC. This water build-up can obstruct active sites, resulting in the decreased performance of Fe-N-CXG-5.8. Nonetheless, the two-stage synthesized catalyst (Fe-N-CXG-5.8) exhibits greater durability due to its less fragile structure. This work highlights the importance of the porous structure in the performance of Fe-N-C catalysts against ORR.

## Figures and Tables

**Table 1:** Textural properties determined from nitrogen physisorption, He pycnometry, and mercury porosimetry.

	S_BET_^a^	V_pore_^a^	V_micro_^a^	V_v_^a,b^	Porosity ^c^	Pore Size nm		
	m^2^ g^−1^	cm^3^ g^−1^	cm^3^ g^−1^	cm^3^ g^−1^	%	Micro	Meso	Macro
CXG-4.5	600	0.26	0.20	0.90	66	1.2 ^a^	-	1880 ^b^
CXG-5.8	666	0.61	0.26	3.04	85	1.2 ^a^	-	145 ^b^
CXG-6	677	1.02	0.27	2.87	82	1.2 ^a^	50 ^a,b^	50 ^a,b^
CXG-7	696	0.80	0.33	0.80	60	1.3 ^a^	7 ^a,b^	-
CXG-8	54	0.02	0.02	0.02	4	-	-	-

^a^ Obtained by N_2_ physisorption. ^b^ Obtained by mercury porosimetry. ^c^ Obtained from V_v_ and the real density.

**Table 2:** Chemical composition of Fe-N-C catalysts determined using EA and ICP.

	ICP (wt.%)	Elemental Analysis (wt.%)		N/C (wt/wt)	N/Fe (wt/wt)
	Fe	C	N		
Fe-N-CXG-4.5	0.95	89.8	0.8	0.0089	0.84
Fe-N-CXG-5.8	0.92	89.1	0.4	0.0045	0.43
Fe-N-CXG-6	0.90	86.9	0.5	0.0058	0.56
Fe-N-CXG-7	0.89	90.2	0.6	0.0067	0.67
Fe-N-CXG-8	1.00	88.4	0.6	0.0068	0.60

**Table 3:** Textural properties determined from N_2_ adsorption/desorption isotherms of Fe-N-CXG-*n*.

	S_BET_	V_pore_	S_micro_	V_micro_	V_mesopore_
	m^2^ g^−1^	cm^3^ g^−1^	m^2^ g^−1^	cm^3^ g^−1^	cm^3^ g^−1^
Fe-N-CXG-4.5	571	0.31	531	0.19	0.12
Fe-N-CXG-5.8	605	0.56	519	0.24	0.32
Fe-N-CXG-6	783	1.20	604	0.26	0.94
Fe-N-CXG-7	891	0.94	464	0.35	0.59
Fe-N-CXG-8	205	0.14	195	0.07	0.07

**Table 4:** Electrochemical parameters for the Fe-N-CXG-*n* catalysts.

Samples	E_onset_	E_1/2_	j_d_	n	Tafel Slope
	V_RHE_	V_RHE_	mA cm^−2^		mV dec^−1^
Fe-N-CXG-4.5	0.73	0.53	−2.66	2.93	102
Fe-N-CXG-5.8	0.77	0.57	−5.16	3.37	98
Fe-N-CXG-6	0.77	0.55	−4.32	3.07	94
Fe-N-CXG-7	0.74	0.52	−3.98	3.41	75
Fe-N-CXG-8	0.53	0.26	−2.11	2.36	173

**Table 5:** Textural properties determined from N_2_ adsorption/desorption isotherms.

	S_BET_	S_micro_	V_pore_	V_micro_
	m^2^ g^−1^	m^2^ g^−1^	cm^3^ g^−1^	cm^3^ g^−1^
Fe-N-OXG-5.8	406	334	0.40	0.15
Fe-N-CXG-5.8	605	519	0.56	0.23

**Table 6:** Chemical composition of Fe-N-C catalysts determined via EA and ICP.

	ICP (wt.%)	Elemental Analysis (wt.%)		
	Fe	C	N	N/C
Fe-N-OXG-5.8	<0.1	97.3	0.72	0.007
Fe-N-CXG-5.8	<0.1	91.3	0.60	0.007

**Table 7:** Chemical speciation of the N1s orbital in atomic %.

	N-Pyridinic	N_x_-M	N-Pyrrolic *	N-Graphitic	N-Oxide	N-Pyridinic/N-Pyrrolic Ratio
Fe-N-OXG-5.8	15.7	12.1	23.2	14.3	34.7	0.68
Fe-N-CXG-5.8	19.1	17.2	18.3	10.7	34.8	1

* N-pyrrolic/pyridonic (for simplicity reasons will be referred to as N-pyrrolic).

**Table 8:** Electrochemical parameters for the Fe-N-OXG-5.8 and Fe-N-CXG-5.8 catalysts.

Samples	E_onset_	E_1/2_	j_d_	n	Tafel Slope
	V_RHE_	V_RHE_	mA cm^−2^		mV dec^−1^
Fe-N-OXG-5.8	0.70	0.44	−3.1	3.1	117
Fe-N-CXG-5.8	0.77	0.57	−5.2	3.4	98

## Data Availability

Data will be available under request.

## Supplementary Materials

The following supporting information can be downloaded at: https://www.mdpi.com/article/10.3390/nano14010014/s1, Figure S1. SEM micrographs of (a) CXG-4.5, (b) CXG-5.8 and (c) CXG-6 at 50,000 magnification and (d) CXG-4.5 at 8000 magnification. Table S1. Chemical composition determined by elemental analysis and ICP. Figure S2. N2-adsorption/desorption isotherms for Fe-N-CXG-5.8 subjected to successive acid leaching/thermal treatments. Table S2. Textural properties determined from nitrogen physisorption isotherms. Figure S3. Polarization curves for the ORR, in RDE at 1600 rpm in O_2_-saturated 0.5 M H_2_SO_4_ for (a) Fe-CXG-6, (b) Fe-N-CXG-4.5, (c) Fe-N-CXG-5.8, (d) Fe-N-CXG-6, (e) Fe-N-CXG-7 (f) Fe-N-CXG-8 subjected to successive cycles of acid leaching/thermal treatments indicated as TTn (n is the number of cycles performed). For clarity reasons, the catalyst without any acid/thermal treatment will be named as pristine and the other samples will be identified only by the final TTn sigla. Figure S4. (a) Koutecky-Levich diagrams obtained at 0.05 V vs. RHE and (b) Tafel plot from LSV of Figure S3c. Table S3. Electrochemical parameters for the Fe-N-CXG-5.8-TTn catalysts. Figure S5. (a) Polarization curves for the ORR, in RDE at 1600 rpm in O_2_-saturated 0.5 M H_2_SO_4_. (b) Koutecky-Levich diagrams obtained at 0.05 V vs. RHE; (c) Tafel plot from LSV at 1600 rpm for the ORR. Table S4. Electrochemical parameters for the CXG-6, Fe-CXG-6 and Fe-N-CXG-6 catalysts. Figure S6. XPS high resolution N1s spectra for (a) Fe-N-OXG-5.8 and (b) Fe-N-CXG-5.8 considering 30% Gaussian and 70% Lorentzian peak shape and Shirley background. Figure S7. Polarization curves for the ORR, in RDE at 400, 600, 900, 1600 and 2500 rpm in O_2_-saturated 0.5 M H_2_SO_4_ of (a) Fe-N-OXG-5.8 and (b) Fe-N-CXG-5.8.
