# Supplementary material for: Tailored Porous Carbon Xerogels for Fe-N-C Catalysts in Proton Exchange Membrane Fuel Cells
Source: Nanomaterials (Basel). 2023 Dec 20;14(1):14. doi: 10.3390/nano14010014 (PMC10780504; doi:10.3390/nano14010014)
Supplement: Supplementary file 1 [file nanomaterials-14-00014-s001.zip › nanomaterials-2743635-supplementary.pdf]

<u>SUPPORTING INFORMATION</u>

Tailored Porous Carbon Xerogels for Fe-N-C Catalysts in Proton Exchange Membrane Fuel Cells

Laura Álvarez-Manuel, Cinthia Alegre*, David Sebastián, Pedro F. Napal, María Jesús Lázaro*

Instituto de Carboquímica, Consejo Superior de Investigaciones Científicas,

50018 Zaragoza, Spain

corresponding authors: cinthia@icb.csic.es, mlazaro@icb.csic.es

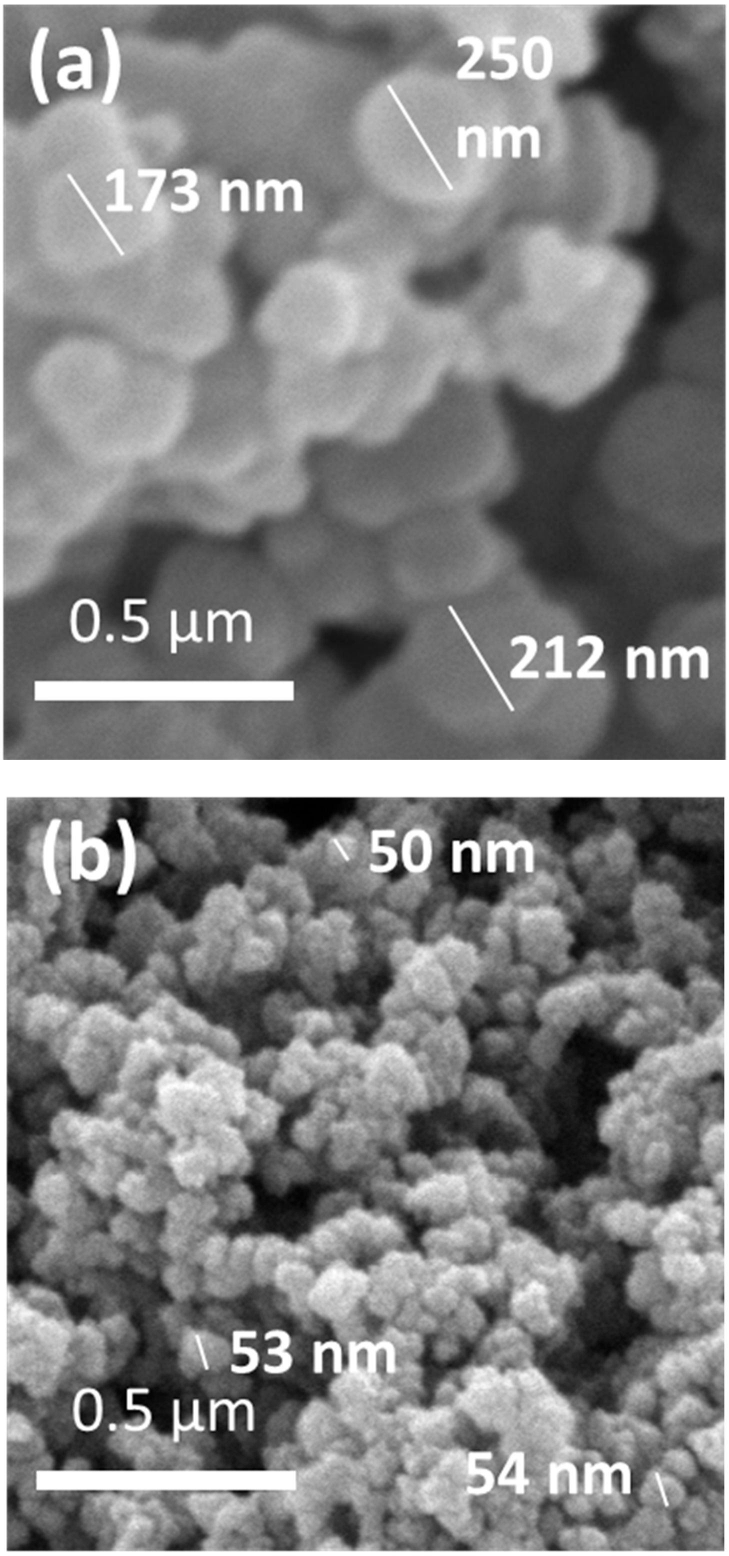
(a)
250 nm
173 nm
0.5 µm
212 nm
(b)
50 nm
53 nm
0.5 µm
54 nm

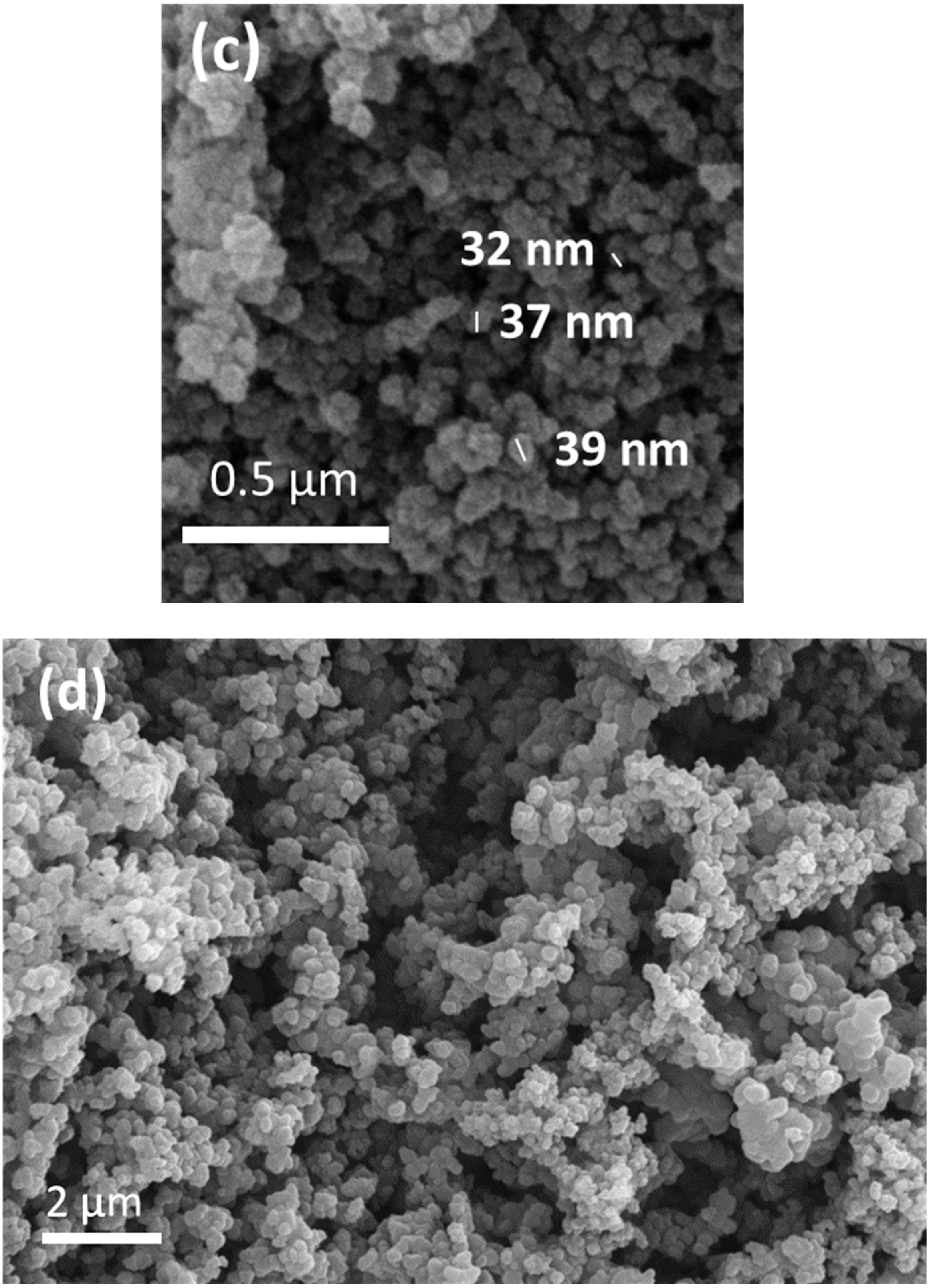

Figure S1. SEM micrographs of (a) CXG-4.5, (b) CXG-5.8 and (c) CXG-6 at 50,000 magnification and (d) CXG-4.5 at 8,000 magnification.

Table S1 first shows the chemical composition of CXG synthesised at pH=6 (CXG-6), Fe-doped CXG-6 without N (Fe-CXG-6) and Fe and N-doped CXG (Fe-N-CXG-6). The presence of N does not affect the amount of Fe introduced [1] as Fe-CXG-6 and Fe-N-CXG-6 have the same amount of Fe. The percentage of Fe present in the catalysts without being subjected to any acid leaching treatment following by a thermal treatment (AL/TT) is similar to the 1 wt% prescribed in the synthesis.

The chemical composition of Fe-N-CXG-5.8 subjected to various AL/TT is also shown in **Table S1**. The Fe-N-CXG catalysts are leached in acid to remove inactive iron particles and then treated at high temperature to be reactivated [2]. The percentage of Fe decrease drastically after the first two (AL/TT). After these treatments the amount of Fe is below the detection limit of the technique. The nitrogen wt%. on the other hand, remains constant and even increases slightly (probably due to the decrease in the percentage of iron), showing that AL/TT do not modify the amount of nitrogen, as already demonstrated in previous works [1, 3].

Table S1. Chemical composition determined by elemental analysis and ICP.

	ICP (wt. %)	Elemental analysis (wt. %)		
	Fe	C	H	N
CXG-6	-	90.8	1.0	-
Fe-CXG-6	0.89	90.7	0.2	-
Fe-N-CXG-6	0.90	86.9	0.5	0.5
Fe-N-CXG-5.8	0.92	89.1	0.8	0.4
Fe-N-CXG-5.8-TT2	0.11	88.7	0.2	0.5
Fe-N-CXG-5.8-TT3	< D.L.*	91.3	0.5	0.6

*below detection limit

The effect on the porous structure of the catalysts was also studied (**Figure S2 and Table S2**). The material resulting from the ball mill mixing of CXG with urea and FeAc (Fe-N-CXG-5.8-BM) prior to pyrolysis was also included in this study. After the first

stage of the synthesis based on the impregnation of the CXG with the N and Fe precursors (urea and iron acetate, respectively) and the milling at 400 r.p.m. of the mixture, only a slight decrease of the surface area is observed (from 666 to 528 m^2 g^{-1}). Therefore, we can conclude that grinding hardly modifies the porous structure of the carbonaceous matrix formed by CXGs. Instead, Prioetti et al. observed significant effects of grinding on the zeolites used as a matrix, since the surface area was reduced from 1800 to 474 m^2 g^{-1} [4]. The main differences in the isotherms, of the catalysts presented in **Figure S2**, are located at low relative pressures, which indicates that the doping process and the treatments affect mainly the micropores. The area and volume of the micropores of the CXG (566 m^2 g^{-1}; 0.26 cm^3 g^{-1}) are reduced both after the first stage of the synthesis Fe-N-CXG-5.8-BM (418 m^2 g^{-1}; 0.21 cm^3 g^{-1}) and after the pyrolysis Fe-N-CXG-5.8 (338 m^2 g^{-1}; 0.19 cm^3 g^{-1}) due to the blockage produced on the micropores by the Fe particles. These iron particles are removed by successive acid leaching [5, 6], allowing the catalysts to recover the area and volume of the micropores, Fe-N-CXG-5.8-TT3 (519 m^2 g^{-1}; 0.24 cm^3 g^{-1}).

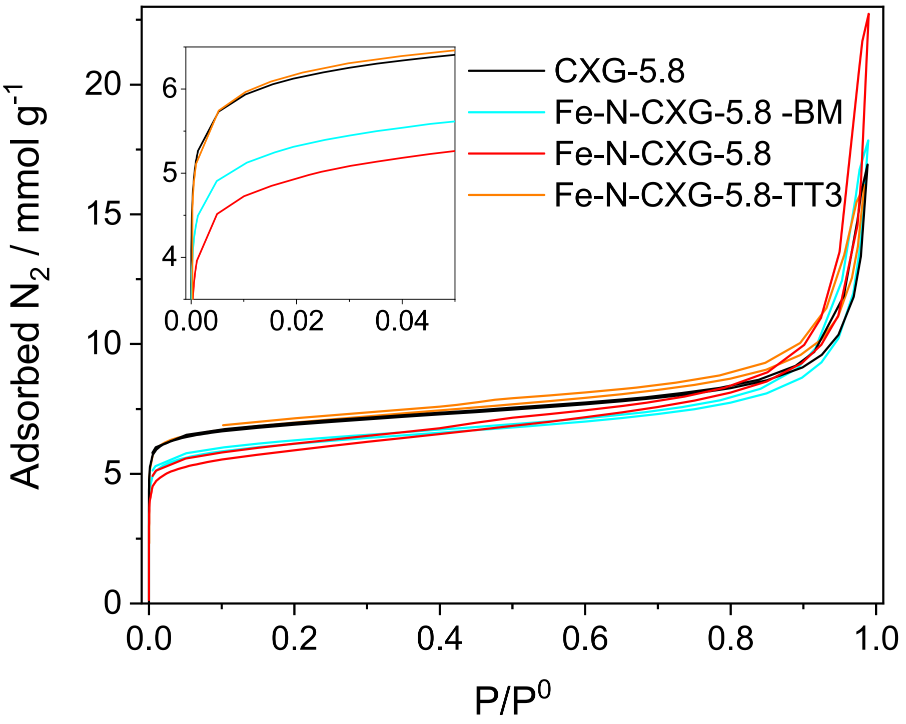

Figure S2. N_2-adsorption/desorption isotherms for Fe-N-CXG-5.8 subjected to successive acid leaching/thermal treatments.

Table S2. Textural properties determined from nitrogen physisorption isotherms

	S_{BET}	V_{pore}	$S_{\mu poros}$	$V_{\mu pore}$	$V_{mesopore}$
	$m^2\ g^{-1}$	*$cm^3\ g^{-1}$*	*$m^2\ g^{-1}$*	*$cm^3\ g^{-1}$*	*$cm^3\ g^{-1}$*
CXG-5.8	666	0.61	566	0.26	0.35
Fe-N-CXG-5.8-BM	528	0.62	418	0.21	0.41
Fe-N-CXG-5.8	496	0.79	338	0.19	0.6
Fe-N-CXG-5.8-TT3	605	0.56	519	0.24	0.32

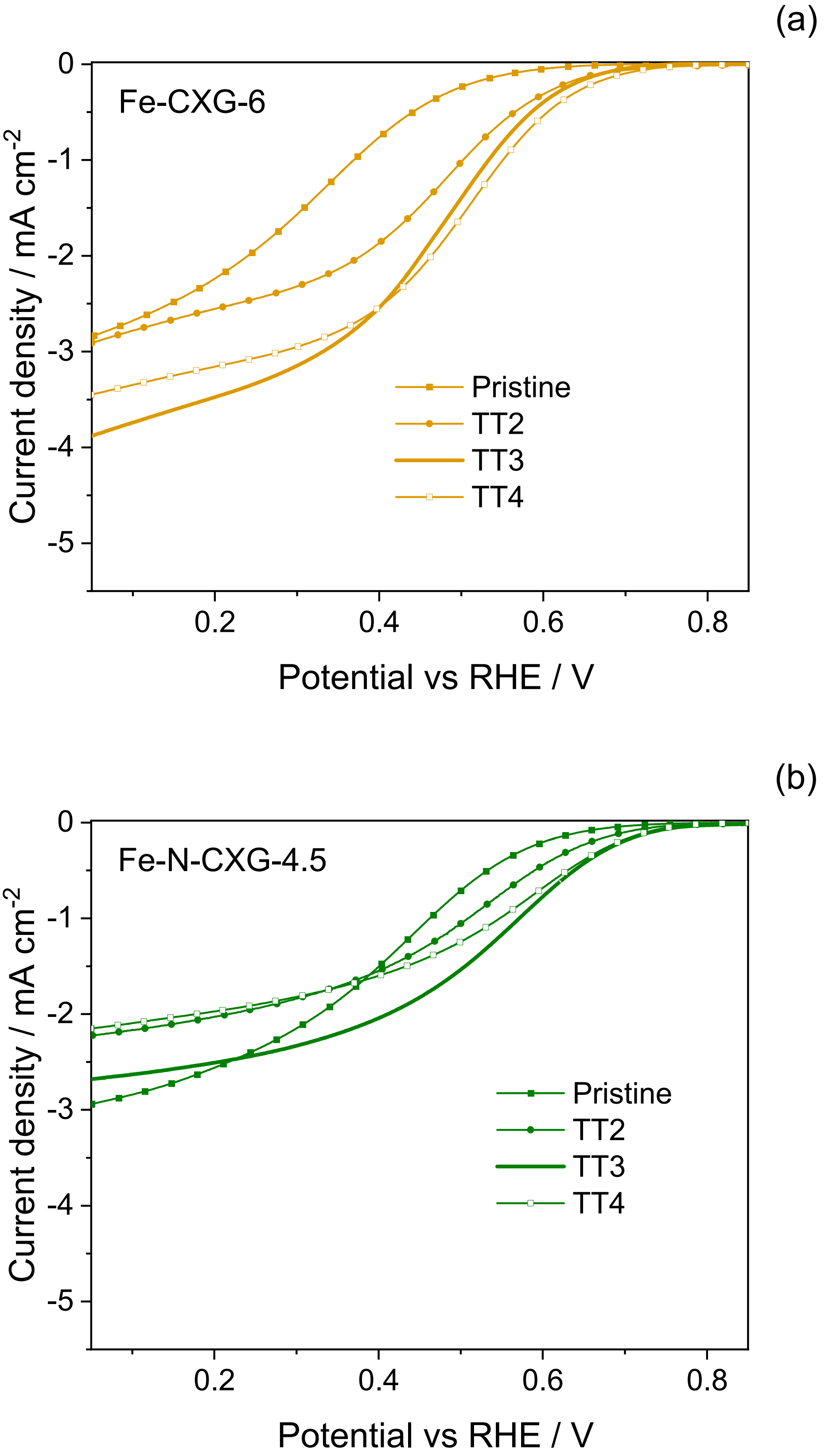
(a)
Fe-CXG-6
Current density / mA cm^{-2}
0
-1
-2
-3
-4
-5
0.2
0.4
0.6
0.8
Potential vs RHE / V
Pristine
TT2
TT3
TT4
(b)
Fe-N-CXG-4.5
Current density / mA cm^{-2}
0
-1
-2
-3
-4
-5
0.2
0.4
0.6
0.8
Potential vs RHE / V
Pristine
TT2
TT3
TT4

(c)

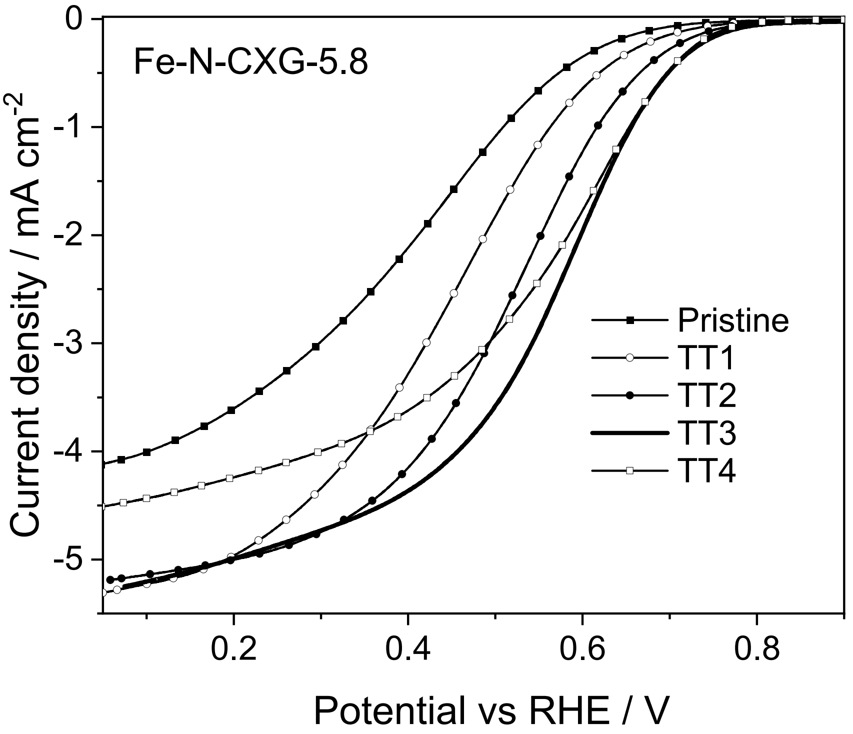

Fe-N-CXG-5.8
Current density / mA cm-2
Potential vs RHE / V
Pristine
TT1
TT2
TT3
TT4
0
-1
-2
-3
-4
-5
0.2
0.4
0.6
0.8

(d)

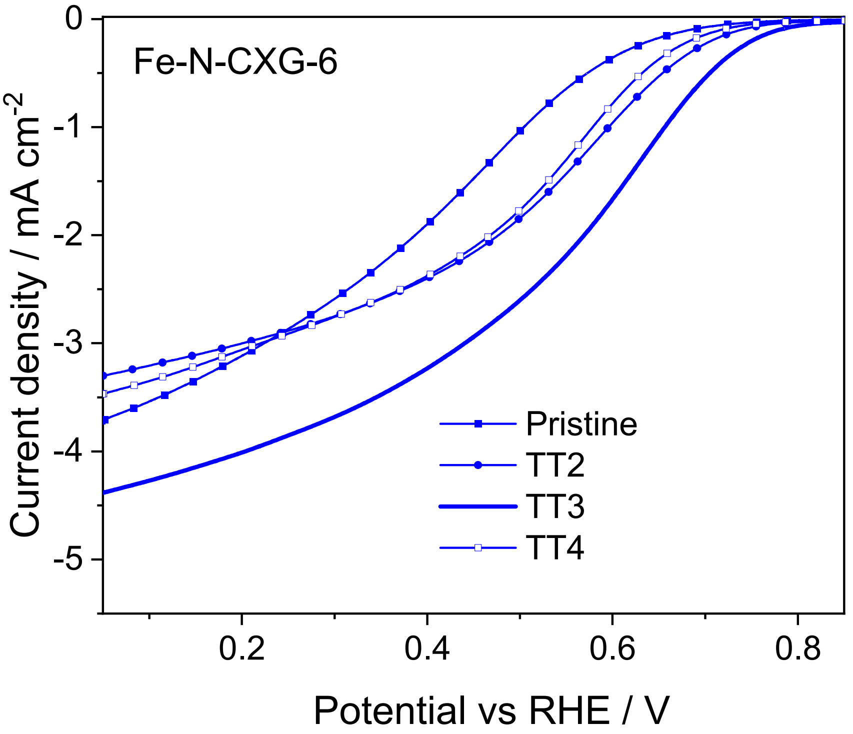

Fe-N-CXG-6
Current density / mA cm-2
Potential vs RHE / V
Pristine
TT2
TT3
TT4
0
-1
-2
-3
-4
-5
0.2
0.4
0.6
0.8

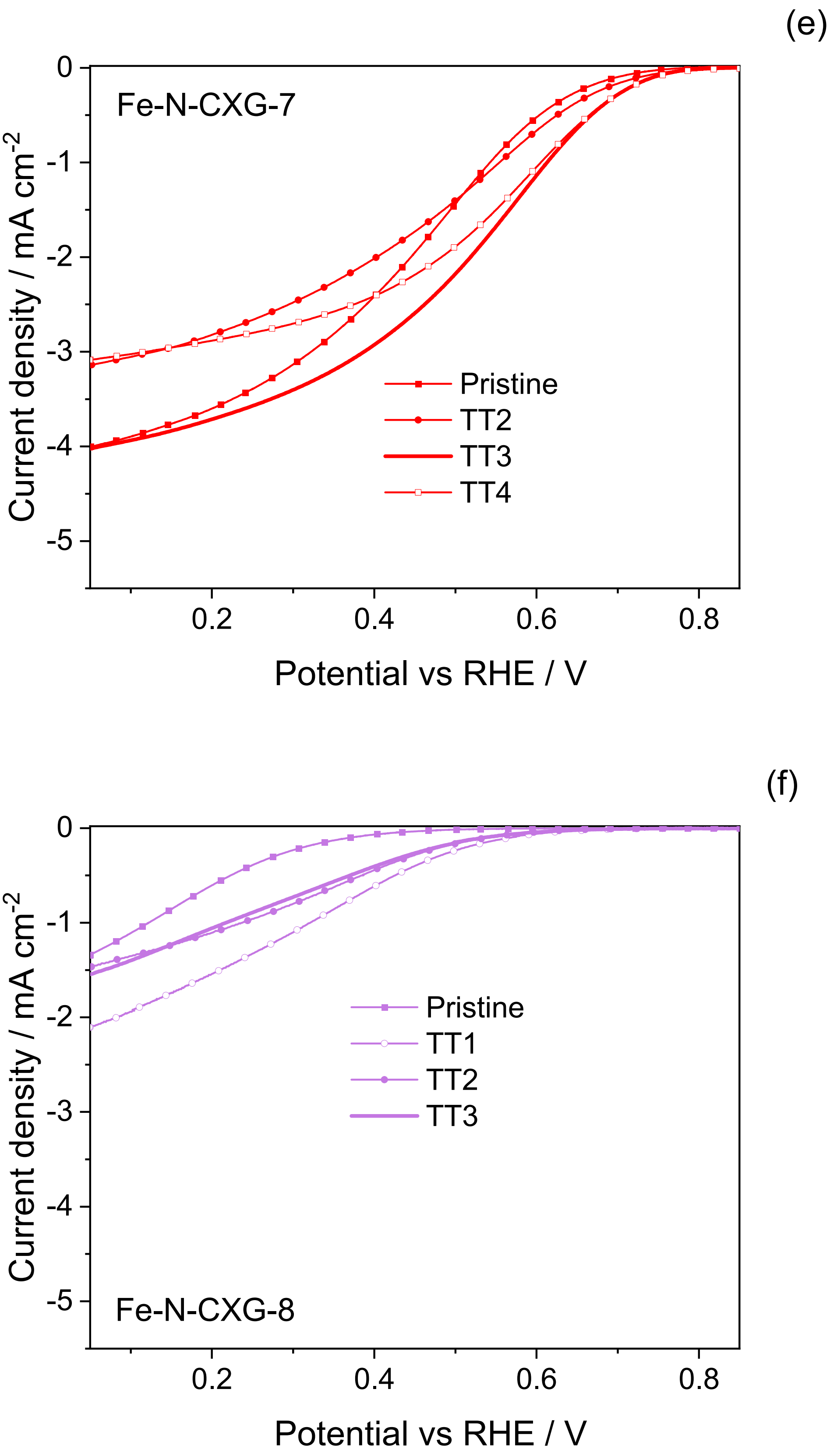

Figure S3. Polarization curves for the ORR, in RDE at 1600 rpm in O_2-saturated 0.5M H_2SO_4 for a) Fe-CXG-6, b) Fe-N-CXG-4.5, c) Fe-N-CXG-5.8, d) Fe-N-CXG-6, e) Fe-N-CXG-7 f) Fe-N-CXG-8 subjected to successive cycles of acid leaching/thermal

treatments indicated as TT*n* (*n* is the number of cycles performed). For clarity reasons, the catalyst without any acid/thermal treatment will be named as pristine and the other samples will be identified only by the final TTn sigla.

Figure S3 c shows the LSV of the catalysts synthesised with CXG-5.8 subjected to a sequence of acid leaching followed by thermal treatment (AL/TT). The electrochemical parameters calculated from the polarisation curves (the onset potential (E_{onset}), the half-wave potentials ($E_{1/2}$) and the limiting current density (j_d)) are compiled in **Table S3**. The values of these parameters increase with the first AL/TT. In a study conducted by Pérez-Rodríguez et al. [7], Fe and N catalysts were synthesized from biomass waste and their activity against the ORR in alkaline media was evaluated. These catalysts underwent acid washes and heat treatments, similar to the ones employed in this work. The findings of their study revealed that the thermal treatments introduced new active sites, enhancing the catalytic activity of the Fe and N catalysts. In this work, the performance of 3 AL/TT led to an improvement in $E_{½}$ of 160 mV. Other authors also demonstrated the positive effect of AL/TT on catalysts prepared on mesoporous carbons [8], on polyaniline-derived carbon black [9] and multiwalled carbon nanotubes [10].

Additional AL/TT (Fe-N-CXG-5.8-TT4) no longer improves the catalytic activity. The Fe-N-CXG-TT3 catalyst presents the optimal amount of iron, providing the maximum number of Fe-N_x-C active sites without blocking the access to the micropores, therefore a number of three acid leaching/thermal treatments for the Fe-N-CXG catalysts will be selected for further investigation. Most of the catalysts studied in this work show optimum electrochemical activity after three AL/TTs.

Figure S4 a represents the Koutecky-Levich (K-L) plot for the different Fe-N-CXG-5.8-TTn catalysts (where n is the number of AL/TT). The number of exchanged electrons is collected in **Table S3.** As occurs with the electrochemical parameters

previously analysed, the catalyst subjected to 3 AL/TT (Fe-N-CXG-5.8-TT3) presents a maximum value of n (n=3.37), corroborating that the development of the Fe-N_x-C active sites is assisted by the AL/TT, since it has been widely demonstrated that in the Fe-N_x-C sites follows a 4e^- ORR mechanism [11]. However, for catalysts subjected to more than 3 AL/TT the number of exchanged electrons begins to decrease. The calculation of the Tafel slope provides information about the reaction kinetics (**Figure S4 b**). **Table S3** shows the Tafel slopes of the Fe-N-CXG-5.8 catalysts subjected to several AL/TT. The Tafel slope decreases with the first AL/TT and the catalyst subjected to 4 AL/TT (Fe-N-CXG-5.8-TT4) has the lower Tafel slope indicating that in this catalyst the ORR proceeds through a more effective pathway.

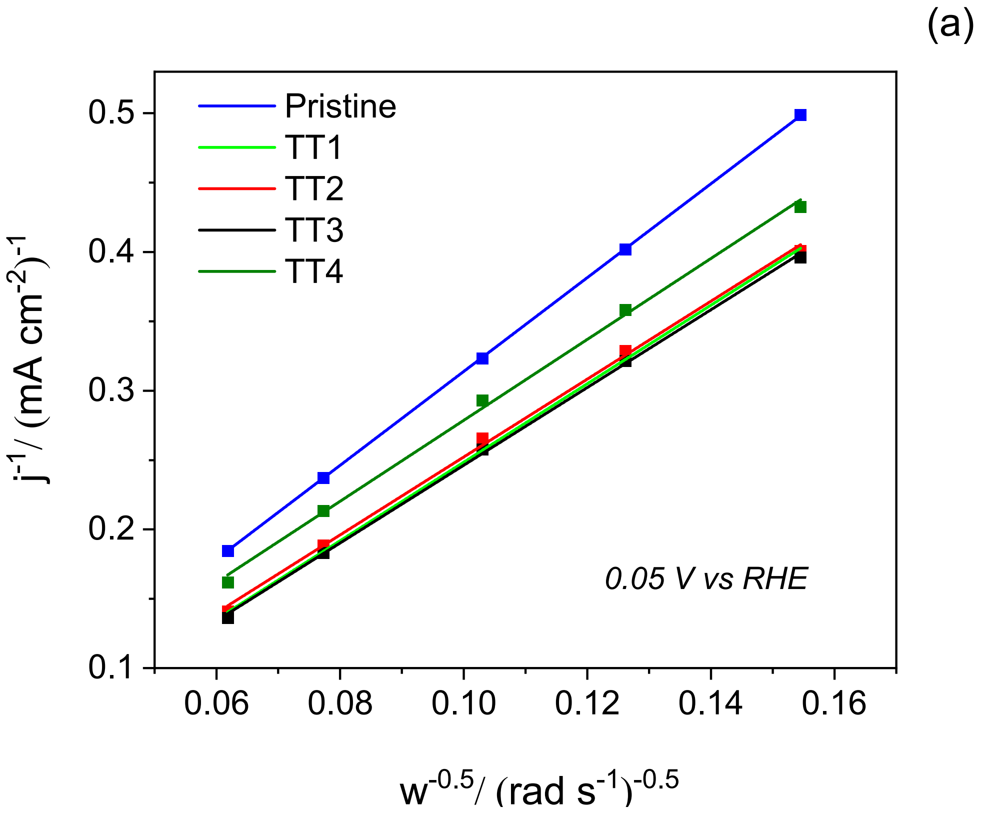

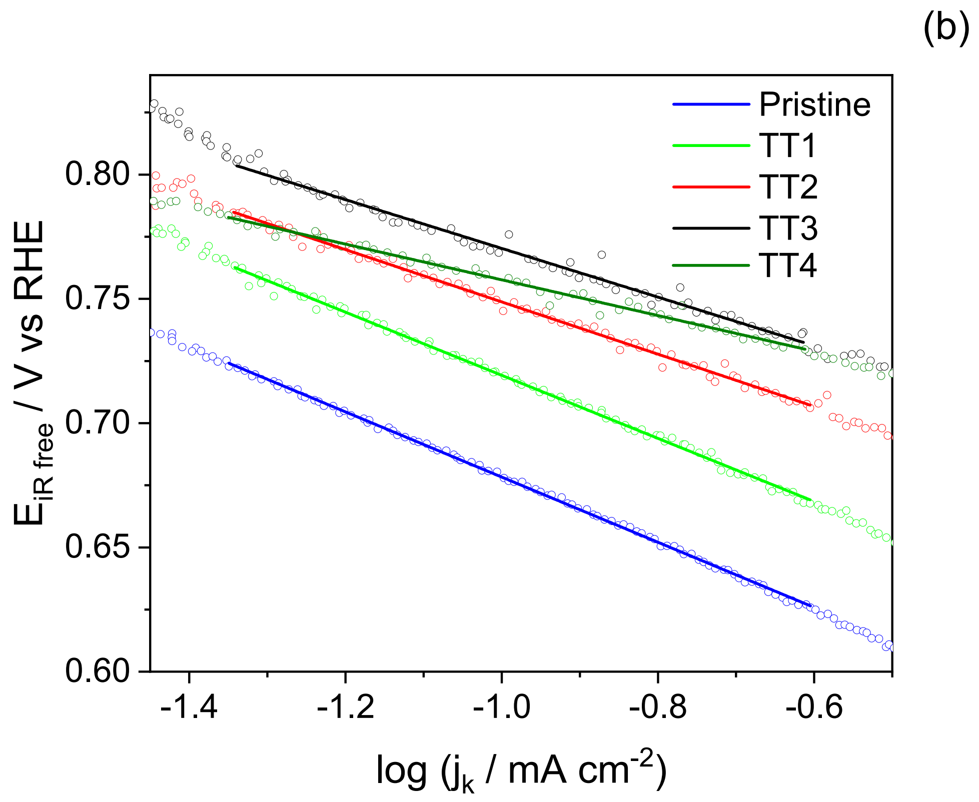

Figure S4. (a) Koutecky-Levich diagrams obtained at 0.05 V vs. RHE and **(b)** Tafel plot from LSV of Figure S3c.

Table S3. Electrochemical parameters for the Fe-N-CXG-5.8-TTn catalysts.

**Samples**	**E_{onset}** V_{RHE}	**$E_{1/2}$** V_{RHE}	**j_d** $mA \cdot cm^{-2}$	**n**	**Tafel slope** $mV\ dec^{-1}$
**Fe-N-CXG-5.8**	0.68	0.41	-4.1	2.79	131
**Fe-N-CXG-5.8-TT1**	0.72	0.44	-5.32	3.33	127
**Fe-N-CXG-5.8-TT2**	0.75	0.52	-5.22	3.36	105
**Fe-N-CXG-5.8-TT3**	0.77	0.57	-5.16	3.37	98
**Fe-N-CXG-5.8-TT4**	0.76	0.57	-4.52	3.23	72

In order to investigate the influence of Fe and N doping, the activity of three different catalysts was measured: an un-doped CXG (CXG-6), a CXG doped solely with Fe (Fe-CXG-6-TT3), and the same CXG doped with both Fe and N (Fe-N-CXG-6-TT3), against the ORR as shown in **Figure S5**. The electrochemical parameters obtained from **Figure S5**, including E_{onset}, $E_{1/2}$, j_d, n, and Tafel slope, are presented in **Table S4**.

The inclusion of Fe in the catalyst clearly enhances its activity, highlighting the significant role of this metal, as confirmed by several previous studies [12, 13]. Furthermore, the incorporation of heteroatoms, such as N, in carbonaceous matrix catalysts has been extensively investigated, as these heteroatoms provide a coordination environment with higher electron density for Fe [14]. Consequently, the Fe-N-CXG-6 catalyst exhibits superior ORR activity compared to Fe-N-CXG-6, further supporting the benefits of Fe and N doping.

(a)

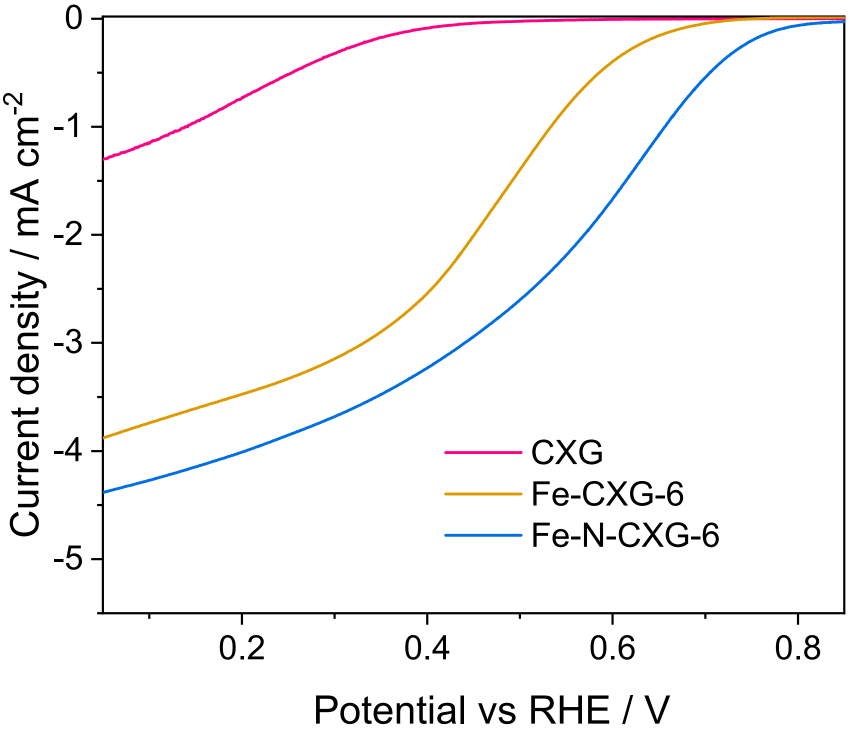

(b)

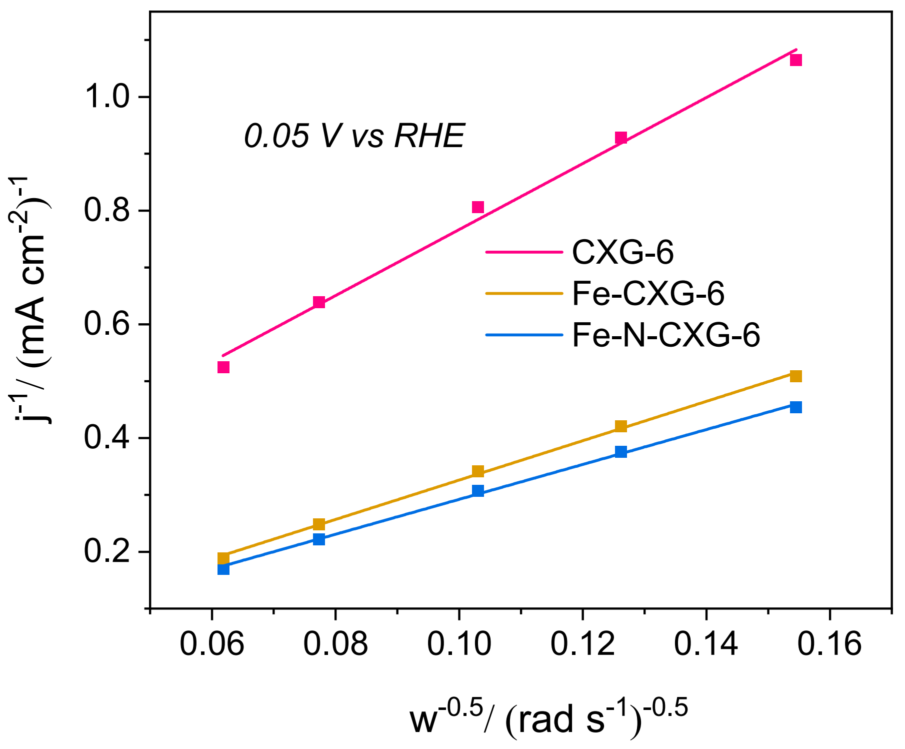

(c)

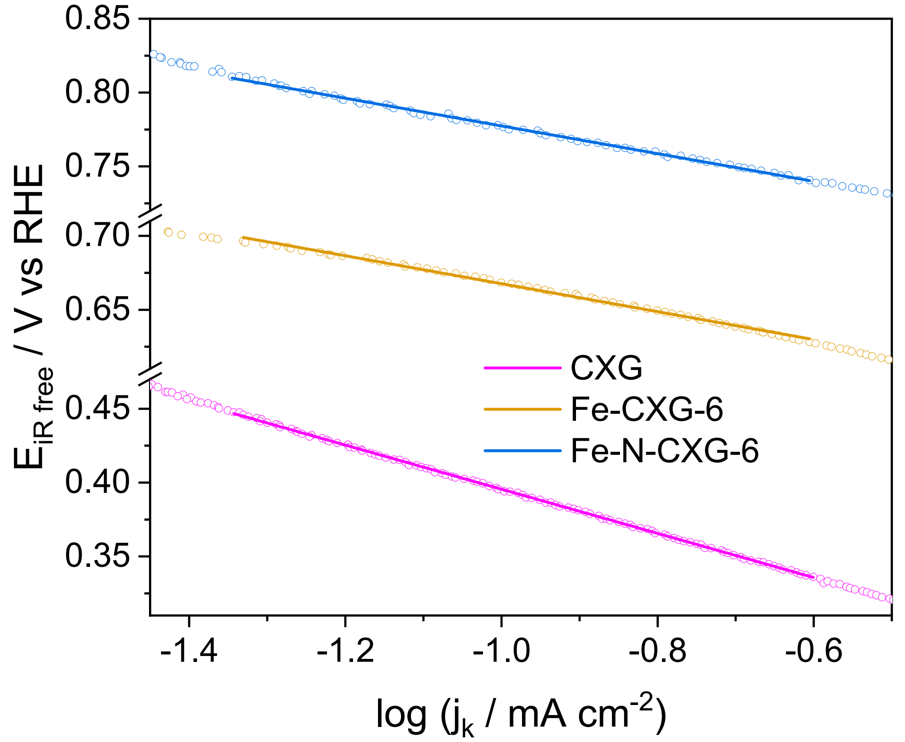

Figure S5. **(a)** Polarization curves for the ORR, in RDE at 1600 rpm in O_2-saturated 0.5M H_2SO_4. **(b)** Koutecky-Levich diagrams obtained at 0.05 V vs. RHE; **(c)** Tafel plot from LSV at 1600 rpm for the ORR.

Table S4. Electrochemical parameters for the CXG-6, Fe-CXG-6 and Fe-N-CXG-6 catalysts.

Samples	E_{onset}	$E_{1/2}$	j_d	n	Tafel slope
	V_{RHE}	V_{RHE}	$mA \cdot cm^{-2}$		$mV\ dec^{-1}$
**CXG-6**	0.39	0.23	-1.23	1.62	149
**Fe-CXG-6**	0.67	0.46	-3.81	2.70	95
**Fe-N-CXG-6**	0.77	0.55	-4.32	3.07	94

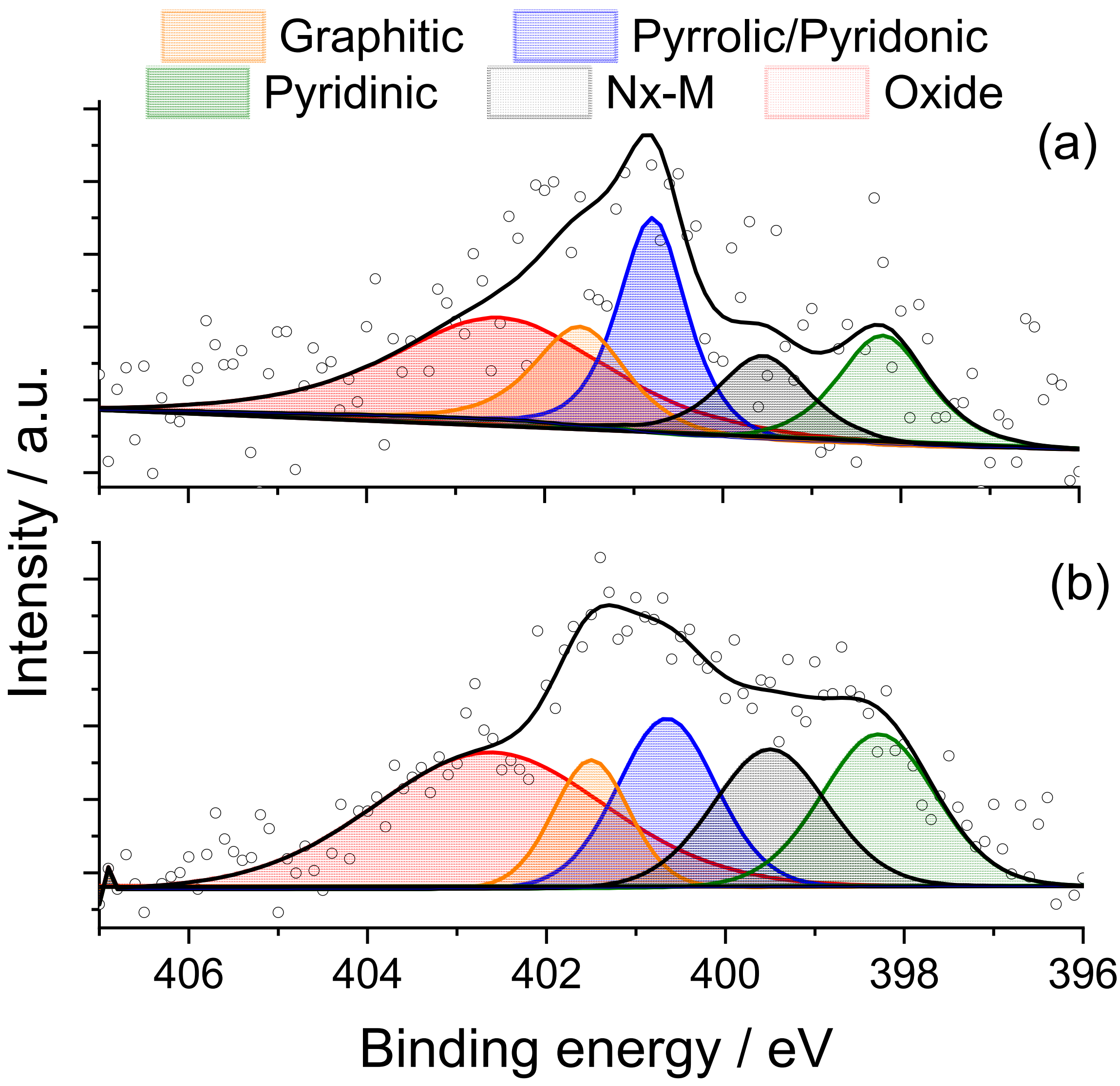

Figure S6. XPS high resolution N1s spectra for (a) Fe-N-OXG-5.8 and (b) Fe-N-CXG-5.8 considering 30 % Gaussian and 70% Lorentzian peak shape and Shirley background.

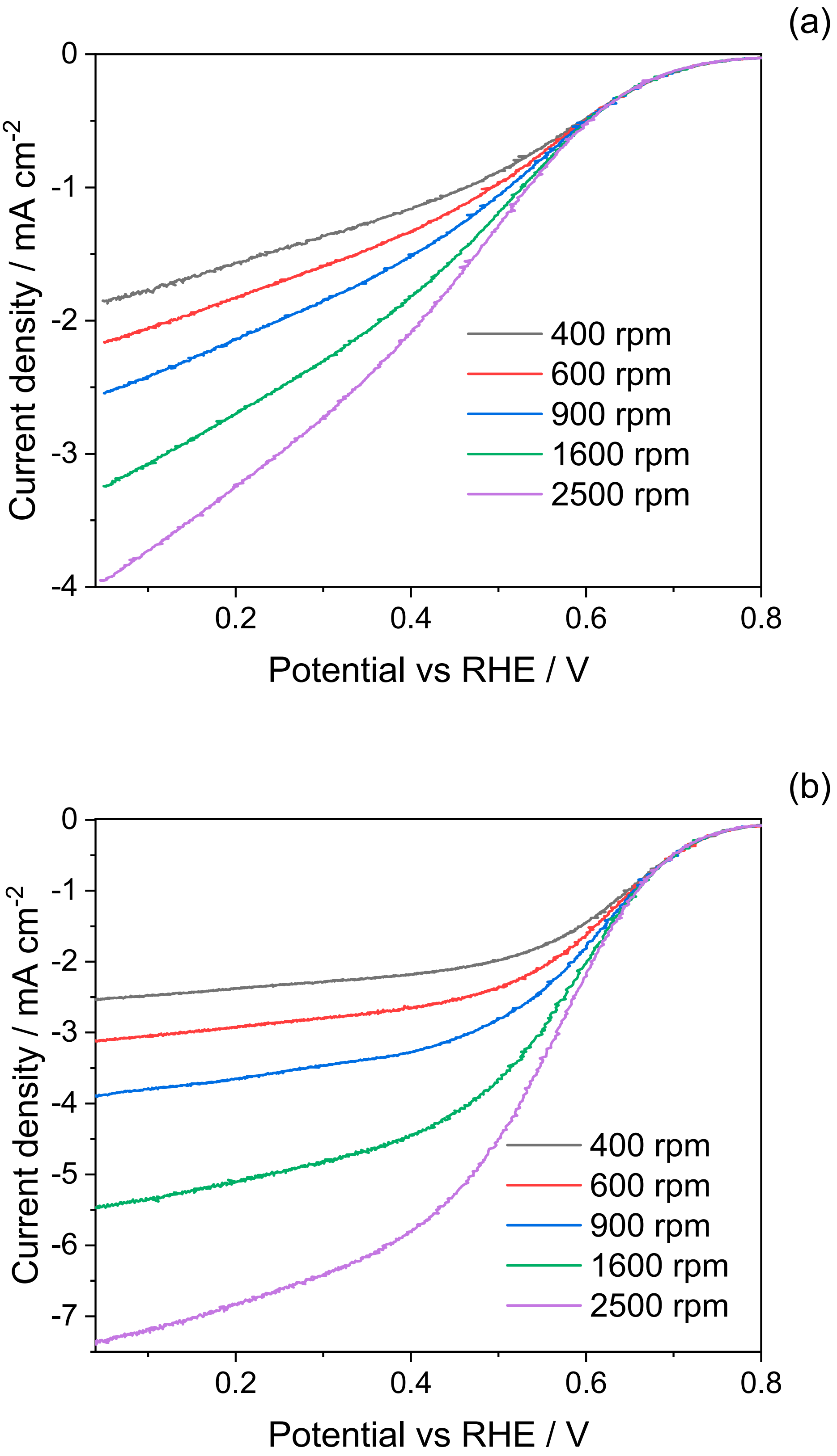

Figure S7. Polarization curves for the ORR, in RDE at 400, 600, 900, 1600 and 2500 rpm in O_2-saturated 0.5M H_2SO_4 of (a) Fe-N-OXG-5.8 and (b) Fe-N-CXG-5.8.
